# Effects of Moso bamboo tubes on color, aroma, physicochemical properties and composition of bitter buckwheat liquor

**DOI:** 10.1038/s41598-025-16966-7

**Published:** 2025-08-27

**Authors:** Juan Du, Kang-Li Yang, Zhi-Qing Yuan, Rui He, Qing Liao, Shu-Juan Liu, Can-Cheng Li, Shou-tong Meng, Xu-yu Long, Qian Su, Pei-ning Zhu

**Affiliations:** 1 School of Art, Changsha Social Work College, Changsha, 410000 China; 2 National & Local Joint Engineering Research Center for Advanced Packaging Material and Technology, School of Packaging and Materials Engineering, School of Packaging Design & Art, Hunan University of Technology, Zhuzhou, 412007 China; 3 Department of Teaching, Zhuzhou Central Hospital, Zhuzhou Affiliated Hospital of Xiangya Medical College, Central South University, Zhuzhou, 412000 China; 4 Jiangxi Agriculture University, Nanchang, 330045 China; 5 Department of Teaching, Zhuzhou Central Hospital, Zhuzhou, 412000 China; 6 National & Local Joint Engineering Research Center for Advanced Packaging Material and Technology, Hunan University of Technology, Zhuzhou, 412007 China

**Keywords:** Bitter buckwheat liquor, Bamboo tube liquor, Color changes, Odor changes, Chemical composition changes, Organic chemistry, Biomaterials

## Abstract

Bamboo tube liquor (BTL) represents a traditional Chinese alcoholic beverage. Although BTL has a long history of folk consumption, the specific compounds that migrate from Moso bamboo into bitter buckwheat liquor (BBL), its effects on liquor quality, and the key chemical markers that characterize BTL remain poorly understood. This study investigated the changes in color, aroma, alcohol and sugar content, physicochemical properties, and chemical composition of BTL after two months of sealed storage in fresh Moso bamboo tubes. The results showed that the color of BTL turned yellow; the pungent odor of NO₂ was significantly reduced; the total sugar content increased from 0.662 to 0.783%; the phenolic content increased to 0.118 g/kg; and the alcohol content decreased to 22.5%. A total of fifty-three new compounds were identified, including those with antioxidant, antibacterial, anti-inflammatory, anti-tumor, and cardiovascular protective effects. These findings confirm the nutritional value and health potential of BTL. This study highlights the unique aging advantages of bamboo tube packaging, offers a scientific basis for its application in functional liquor production, and suggests new directions for the high-value utilization of Moso bamboo.

**Supplementary Information:**

The online version contains supplementary material available at 10.1038/s41598-025-16966-7.

## Introduction

Bamboo tube liquor (BTL) is a traditional alcoholic beverage in China, widely consumed among ethnic minority groups, particularly the Dai people in Yunnan Province, the Hakka people in Fujian and Zhejiang Provinces, and the Tujia people in Hunan and Guizhou Provinces. It holds an important position in Chinese Baijiu culture. The distinctive feature of BTL is that bitter buckwheat liquor (BBL) is infused into the Moso bamboo through micro-perforation technology, and after being sealed and aged for 1–3 months, it becomes available. During this period, BBL stored in Moso bamboo tubes absorbs nutrients from the bamboo, thereby enhancing its health benefits and improving its flavor^1^. Selecting an appropriate container can exert a positive influence on the alcoholic beverage stored within it. For example, Wei et al. reported that, compared with stainless steel storage, storage in ceramic vessels can increase the content of beneficial trace elements in baijiu and further enhance its flavor^2^.

Currently, there are approximately 1,700 bamboo species in the world. Moso bamboo has fast growth, high yield, early maturity and a wide range of uses, accounting for more than two-thirds of the total bamboo forest area in China. After deep processing, it is used in construction, furniture, and paper, and can also be used as an extract in the pharmaceutical, food, and chemical industries^3,4^. In addition, Moso bamboo stalks can reach a height of up to 20 m, with a diameter usually ranging from 5 to 10 cm, a wall thickness of about 1 cm, and an internode length of 20–30 cm—making it the most suitable and commonly used bamboo species for producing BTL^5^. Research has shown that bamboo culms and leaves are rich in organic acids, polysaccharides, mineral elements, flavonoids and other chemical components. Although BTL has a long history in Chinese folk culture, the specific components that migrate from Moso bamboo into bitter buckwheat liquor (BBL) during the aging process, their effects on the quality of BBL, and the characteristic compounds of BTL remain largely unexplored. Few studies have systematically addressed these issues. In this study, a combination of techniques—including alcohol content analysis, electronic nose detection, CIELab colorimetry, total sugar and phenolic content assays, total flavonoid detection, and LC-MS—was employed to investigate the color, aroma, alcohol content, sugar content, physicochemical properties, and chemical composition of BTL. The beneficial components and health-related functions of BTL were examined and interpreted from a scientific perspective.

## Conclusion

After being sealed and brewed in fresh Moso bamboo tubes for two months, the color of BTL turned yellow, the pungent odor of NO₂ was greatly reduced, the sweetness of the liquor body increased, the alcohol content decreased, and fifty-three new compounds were identified. Among these, nine exhibited antioxidant activity, seven had antibacterial properties, fourteen showed anti-inflammatory effects, six were enzyme inhibitors, twelve demonstrated anti-tumor potential, five exhibited lipid-lowering activity, three functioned as nutritional supplements, one had blood pressure-lowering properties, two possessed anti-fatigue effects, and two were associated with cardiovascular and cerebrovascular protection. In addition, compared to BBL, the relative concentrations of nineteen beneficial compounds in BTL significantly increased, while those of three harmful substances markedly decreased. These results indicate that BTL is rich in bioactive compounds and potentially beneficial to human health. This study highlights the functional advantages of Moso bamboo tube as a natural aging container over conventional packaging materials. It provides scientific support for the rational development of functional alcoholic beverages and offers a foundation for further research on the nutritional composition of BTL. Moreover, it presents a new perspective on the value-added utilization of Moso bamboo and promotes its innovative applications.

## Discussion

### Color changes

**Fig. 1:**
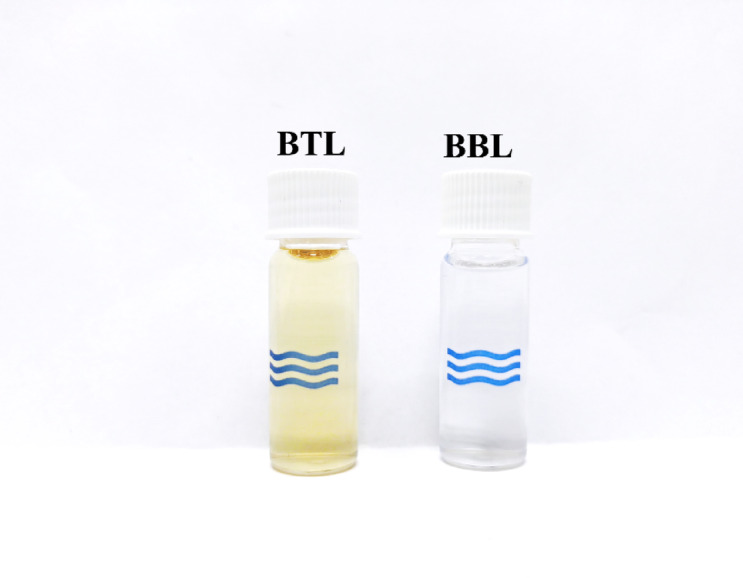
Physical Images of BTL (left) and BBL (right).

After being sealed and brewed in Moso bamboo tubes, BTL undergoes a transformation from a transparent liquid to a vibrant yellow hue (Fig. 1). The color parameters of BTL are subsequently calculated using the CIELab system. In CIELab analysis, L^*^ represents lightness, where L^*^ = 0 indicates black and L^*^ = 100 indicates white (or maximum brightness). The a^*^ value represents the red-green axis, with a^*^ > 0 indicates a tendency toward red and a^*^ < 0 indicates a tendency toward green. The b^*^ value corresponds to the yellow-blue axis, where b^*^ > 0 indicates a tendency toward yellow and b^*^ < 0 indicates a tendency toward blue. The calculation formulas are as follows:

1 $$\:{\text{L}\text{*}=116(\frac{Y}{{Y}_{0}})}^{\frac{1}{3}}-16$$

2 $$\:{\text{a}\text{*}=500\:[(\frac{X}{{X}_{0}})}^{\frac{1}{3}}-{(\frac{Y}{{Y}_{0}})}^{\frac{1}{3}}]$$

3 $$\:{\text{b}\text{*}=200[(\frac{Y}{{Y}_{0}})}^{\frac{1}{3}}-{(\frac{Z}{{Z}_{0}})}^{\frac{1}{3}}]$$

$$\:\text{S}\text{*}=\sqrt{{\left({a}^{*}\right)}^{2}+{\left({b}^{*}\right)}^{2}}$$

4 $$\:\text{X}\hspace{0.17em}=\hspace{0.17em}14.172\text{T}_{440}\hspace{0.17em}+\hspace{0.17em}28.583\text{T}_{530}\hspace{0.17em}+\hspace{0.17em}52.727\text{T}_{600}-0.462$$

5 $$\:\text{Y}\hspace{0.17em}=\hspace{0.17em}9.005\text{T}_{440}\hspace{0.17em}+\hspace{0.17em}62.965\text{T}_{530}\hspace{0.17em}+\hspace{0.17em}28.168\text{T}_{600}\hspace{0.17em}-\hspace{0.17em}0.063$$

6 $$\:\text{Z}\hspace{0.17em}=\hspace{0.17em}94.708\:\text{T}_{440}\hspace{0.17em}+\hspace{0.17em}15.889\:\text{T}_{530}\hspace{0.17em}-\hspace{0.17em}5.233\:\text{T}_{600}\hspace{0.17em}+\hspace{0.17em}1.777$$

7 $$\:\text{X}_0\hspace{0.17em}=\hspace{0.17em}97.29,\quad\:\text{Y}_0\hspace{0.17em}=\hspace{0.17em}100,\quad\:\text{Z}_0\hspace{0.17em}=\hspace{0.17em}116.14$$

$$\:\text{T}_\text{i}=10^{-\hspace{0.17em}\text{A}},\quad\:\text{i}\hspace{0.17em}=\hspace{0.17em}\text{440,530,600}$$.

Where A refers to the absorbance at each wavelength.

**Table 1:** CIELab parameter values for BBL and BTL samples.

Sample	L^*^	a^*^	b^*^	S^*^
BBL	100	0	0	0
BTL	97.737	−2.5	15.2	15

After being stored in 1-year-old Moso bamboo tubes for 2 months, the color changes of BBL are shown in Table 1. The L^*^ value of BTL decreases from 100 to 97.737, indicating a decrease in brightness. The a^*^ value decreases from 0 to −2.5, indicating a shift toward green, while the b^*^ value increases from 0 to 15.2, indicating a pronounced shift toward yellow. This color transformation is primarily attributed to the migration of specific flavonoids, particularly Vicenin II and Vicenin III, from the Moso bamboo tube into BBL. These compounds, structurally similar to the C-glycosyl flavonoid Vicenin II, exhibit strong ultraviolet-visible light absorption and are likely responsible for the observed yellow coloration and decreased brightness (L^*^) of the liquor^6–9^. Notably, the change in b^*^ is more pronounced than that of L^*^ and a^*^, indicating that yellow is the dominant chromatic shift in BTL. In addition to flavonoids, the decrease in L^*^ may also be attributed to the diffusion of polysaccharides from the bamboo into BBL. These high-molecular-weight compounds can increase the turbidity of the liquor and scatter incident light, thereby lowering its brightness. This phenomenon is likely driven by physicochemical interactions—such as hydrogen bonding and colloidal aggregation—between bamboo-derived macromolecules and liquor constituents^10,11^. As further evidence, the increase in total sugar content in BTL, as shown in Table 2, supports the presence of dissolved polysaccharides contributing to the observed turbidity and brightness reduction.

### Odor changes

**Table 2:** The name and main performance of each sensor in electronic nose (PEN3).

The number of sensors	Name	Sensor performance description	Gas reference
S1	W1C	sensitive to aromatic compounds	Methylbenzene, 10 ppm
S2	W5S	broad-spectrum response, extremely sensitive to nitrogen oxides	NO_2_, 1 ppm
S3	W3C	sensitive to ammonia and aromatic compounds	Benzene, 10 ppm
S4	W6S	sensitive to hydrogen gas	H_2_, 100 ppm
S5	W5C	sensitive to olefins, aromatic compounds, and non-polar compounds	Propane, 1 ppm
S6	W1S	sensitive to methane	CH_3_, 100 ppm
S7	W1W	sensitive to sulfur-containing compounds and some aromatic compounds	H_2_S, 1 ppm
S8	W2S	sensitive to alcohols and some aromatic compounds	CO, 100 ppm
S9	W2W	sensitive to sulfur-containing and aromatic compounds	H_2_S, 1 ppm
S10	W3S	sensitive to alkanes	Methane, 100 mg/kg

**Fig. 2:**
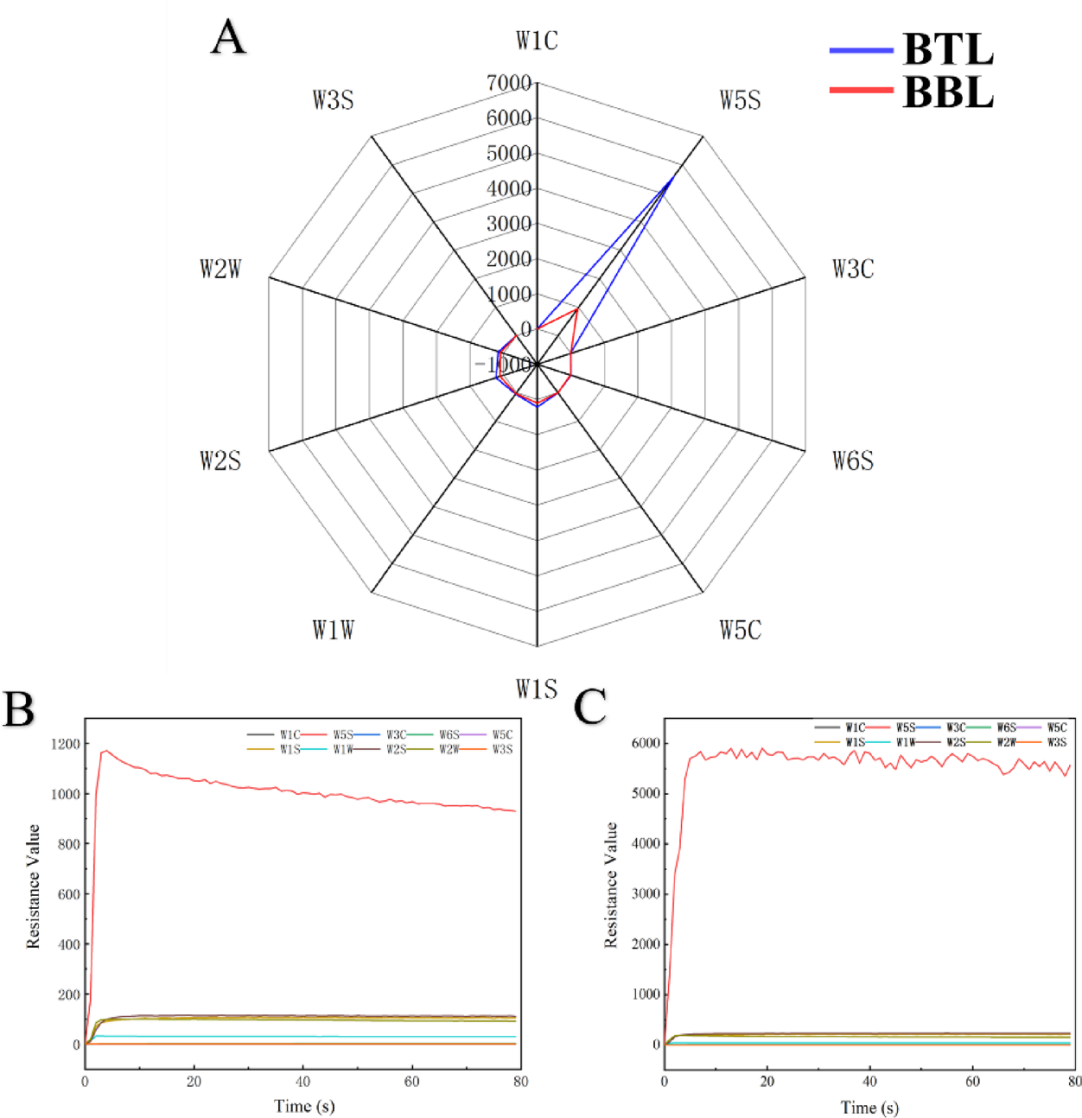
**A** depicts the 70th-second response diagrams of BBL and BTL corresponding to the sensor; **B** illustrates the electronic nose sensor response diagram of BBL; and **C** displays the electronic nose sensor response diagram of BTL.

After two months of sealed aging in Moso bamboo tubes, the aroma of BBL has undergone significant changes. The aromatic characteristics of BTL were measured using an electronic nose. Table 2 displays the gas responses of 10 sensors, while Fig. 2B and C display the corresponding sensor response curves for BBL and BTL, respectively. Each curve represents the change in electrical resistance over time as volatile components pass through the sensor array. Initially, the resistance ratio is low; as volatile substances accumulate on the sensor surfaces, the resistance gradually increases and eventually stabilizes. In Fig. 2B, the resistance values for BBL show that sensors W1C, W3S, W5C, W6S, and W3C maintain resistance values of 0, while W1W shows near-zero resistance. Sensors W2W, W2S, and W1S stabilize around 100 after the third second. Notably, W5S exhibits the highest resistance, decreasing from approximately 1180 to 927. In Fig. 2C, BTL exhibits response patterns comparable to those of BBL for W1C, W3S, W5C, W6S, and W3C, while the responses of W1W, W2W, W2S, and W1S remain low. However, W5S shows a markedly higher resistance, fluctuating between 5500 and 6000. For analysis, the 70th second was selected as the reference point, given the stabilization of sensor responses at that time.

As shown in Fig. 2A, the five sensors (W1C, W3S, W5C, W6S, and W3C) show no response variation between BBL and BTL. Compared to BBL, W2W, W1W, W1S, and W2S in BTL exhibit slight decreases, indicating a reduction in sulfur-containing compounds, methyl compounds, alcohols, and aldehydes/ketones, respectively (Tables 2 and 3). This observation is consistent with previous findings that plant-based aging vessels can reduce low-molecular-weight alcohols and sulfur compounds through adsorption and chemical transformation (Tomasz et al., 2020), thereby improving aroma quality^12^. Moreover, the resistance of W5S in BTL significantly decreases—from 5599 to 952 (an 83% reduction)—suggesting a substantial decline in nitrogen oxides. Given the high sensitivity of W5S to NO₂, and the known presence of such irritants in low-quality Baijiu, this finding implies that bamboo aging helps reduce harmful volatile gases.

Taken together, the results indicate that Moso bamboo tubes not only serve as storage containers but also act as active modulators of volatile profiles. This is likely achieved through physical adsorption, pore-based retention, and possible chemical interactions with bamboo-derived constituents. Such mechanisms contribute to reducing undesirable volatiles and enhancing the overall aroma of the final product.

### Physicochemical properties

**Table 3:** Physicochemical properties of BBL and BTL.

Sample	Alcohol concentration (% vol)	Total sugar content (%)	Total flavonoid content (%)	Total phenolic content(g/kg)
BBL	42.0	0.662	non-detectable	non-detectable
BTL	22.5	0.783	non-detectable	0.118

The physicochemical properties of alcohol are fundamental quality indicators, influencing attributes such as color, taste, and nutritional composition. Table 3 presents the physicochemical properties of BTL and BBL. As shown in Table 3, after two months of sealed aging, the alcohol content of BBL decreased significantly from 42% vol to 22.5%, representing a 46.4% reduction. This reduction may be attributed to the Moso bamboo tube absorbing a substantial amount of ethanol, providing a natural and effective means of reducing alcohol content.

Moso bamboo poles are rich in polysaccharides and nutrients (phenols, flavonoids, amino acids, etc.). During the aging process, polysaccharides from Moso bamboo leach into BBL. As outlined in Table 3, the total sugar content of BBL increased from 0.662 to 0.783% after two months of closed aging, reflecting an 18.2% increase. As shown in Tables 4, 16 new glycoside compounds were identified in BTL.

Total flavonoid testing showed that neither BBL nor BTL contained flavonoid levels below the detection limit. However, LC-MS testing revealed the presence of 9 new flavonoids and flavonoid compounds in BTL. It is possible that the flavonoid content in BTL is lower than the detection limit of the method.

Total phenolic testing revealed an increase in BTL from 0 to 0.118 g/kg. This finding is corroborated by the analysis results of LC-MS. Compared with BBL, BTL contained three additional phenolic compounds, likely derived from the dissolution of hemicellulose in Moso bamboo during brewing. The newly identified compounds were 2-methoxy-4-[3-[3-(trifluoromethyl)anilino]imidazo[1,2-a]pyrimidin-2-yl]phenol, 9-O-feruloyl-5,5′-dimethoxylariciresinol, and (–)-syringaresinol di-O-glucoside.

### Chemical composition

**Fig. 3:**
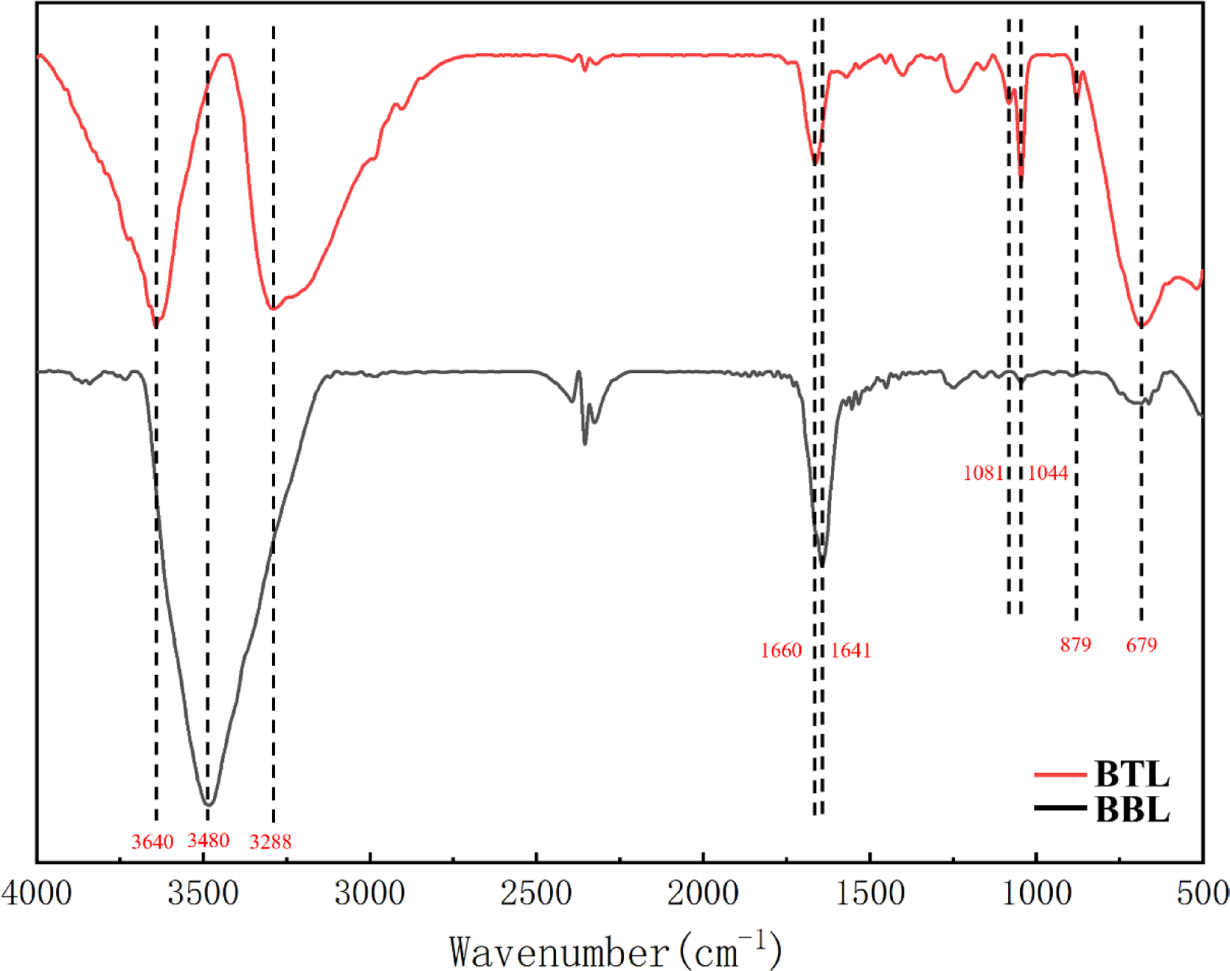
FT-IR diagrams of BTL and BBL.

As depicted in Fig. 3, the peak at 3480 cm^−1^ in BBL may indicate the stretching vibration of intermolecular -OH in alcohols, while the peaks at 3640 cm^−1^ and 3288 cm^−1^ in BTL may correspond to a transformation from intermolecular -OH in alcohols to free -OH in flavonoids, glycosides and terpenoids. This transformation could be attributed to the exchange of substances between the -OH functional group of BBL and the nutrients in fresh Moso bamboo tubes during sealed brewing. The peak at 1641 cm^−1^ likely represents the stretching vibration of carbonyl groups (C = O) in flavonoids, glycosides, quinones, and terpenoids. After sealed brewing, this peak shows a noticeable shift. BTL introduces additional peaks at 1081 cm^−1^ and 1044 cm^−1^, possibly due to the C-O stretching vibration of alcohols and phenols. The observed alteration in total phenolic content in Table 3 further supports this interpretation. Additionally, BTL exhibits an added peak at 879 cm^−1^, likely arising from the stretching vibration of glycosidic bonds. Furthermore, the peak at 679 cm⁻¹ may be attributed to the out-of-plane bending vibration of C–H bonds.

### Analysis of newly added substances in BTL

LC-MS was employed to detect the components of BBL and BTL in order to investigate the influence of Moso bamboo tubes as brewing containers on the chemical composition of BBL. A total of 248 compounds were identified in BBL, and 592 in BTL, considering only those with a fragmentation score ≥ 80. The detected compounds in BBL and BTL were compared, and the newly identified compounds in BTL were summarized in Table S2. Table 4 classifies the compounds listed in Table S2 (fragmentation score ≥ 80) according to their biological functions. A total of 53 new compounds were identified in BTL after two months of sealed brewing. These include 6 flavonoids, 3 isoflavonoids, 9 amino acids, 3 phenolic compounds, 8 terpenoids, 16 glycosides, 3 quinones, and 5 other compounds.

**Table 4:** The nutritional value assessment of newly detected compounds in BTL.

No.	Identification	antioxidant properties	Antibacterial properties	Anti-inflammatory properties	Inhibitor properties	Antitumor properties	Lipid lowering properties	Nutrients	Lower blood pressure properties	Resist fatigue properties	Anti cardiovascular and cerebrovascular diseases	References
**Flavonoids**												
1	Vicenin II	√	/	√	√	√	√	/	/	/	/	^9,13,14^
2	Vicenin III	√	/	√	/	/	/	/	/	/	/	^6^
3	Calealactone B	/	/	/	/	√	/	/	/	/	/	^15^
4	Epmedin C	/	/	/	/	/	√	/	/	/	/	^16^
5	Jacein	/	√	/	/	√	/	/	/	/	/	^17,18^
6	Yukovanol	/	/	/	/	/	/	/	/	/	/	/
**Isoflavonoids**												
7	Phomopsinone D	/	√	/	/	/	/	/	/	/	/	^19^
8	Isoschaftoside	/	√	√	√	/	√	/	/	/	/	^20,21^
9	Norwogonin-8-O-glucuronide	/	/	/	/	/	/	/	/	/	/	/
**Amino acid**												
10	D-Tryptophan	/	√	/	/	√	/	√	/	/	/	^22–26^
11	Glycyltyrosine	/	/	/	/	√	/	√	/	/	/	^27,28^
12	Ala Glu Leu	/	/	/	/	/	/	/	√	/	/	^29^
13	Gly Leu	√	√	/	/	√	/	√	/	/	/	^30–33^
14	Asp Ile Phe	/	/	/	/	/	/	/	/	/	/	/
15	N-Acetyl-tryptophan	√	/	√	√	/	/	/	/	/	/	^34–36^
16	Ser Leu Ile	/	/	/	/	/	/	/	/	/	/	
17	Asp Phe		/	/	/	/	/	/	/	√	/	^37^
18	Ile Leu Glu	/	/	/	/	/	/	/	/	/	/	/
**Phenolic compounds**												
19	2-methoxy-4-[3-[3-(trifluoromethyl) anilino imidazo [1,2-a] pyrimidin-2-yl] phenol	/	/	/	/	/	/	/	/	/	/	/
20	9-O-Feruloyl-5,5’-dimethoxylariciresinol	/	/	√	/	√	/	/	/	/	/	^38,39^
21	(-)-Syringaresinol di-O-glucoside	√	/	/	/	/	/	/	/	/	/	^40^
**Terpenoids**												
22	Fibleucin	/	/	√	/	/	/	/	/	/	/	^41^
23	Morindin	/	/	/	/	/	/	/	/	/	/	/
24	Secologanic acid	/	/	√	/	/	/	/	/	/	/	^42^
25	Paeonilactone C	/	/	/	/	/	/	/	/	/	/	/
26	Oleuropein aglycone	√	/	√	√	√	√	/	/	/	√	^43,44^
27	(+)-Abscisic acid	/	√	/	/	/	/	/	/	/	/	^45^
28	Ciwujiatone	√	/	√	/	/	/	/	/	/	/	^46^
29	Zeylenol	/	/	√	/	√	/	/	/	/	/	^47^
**Glycosides**												
30	Fraxamoside											
31	Torosachrysone 8-O-beta-gentiobioside	/	/	/	/	/	/	/	/	/	/	/
32	Forsythenside A	/	/	/	/	√	/	/	/	/	/	^48^
33	Sibiricose A5	√	/	/	/	/	/	/	/	/	/	^49^
34	3-Deoxyguanosine	/	/	/	/	/	/	/	/	/	/	/
35	N2, N2-Dimethylguanosine	/	/	/	/	/	/	/	/	/	/	/
36	Eugenol gentiobioside	/	/	/	/	/	/	/	/	/	/	/
37	Acanthoside B	√	/	√	/	/	/	/	/	√	/	^50^
38	Orcinol gentiobioside	/	/	/	/	/	/	/	/	/	/	/
39	Adenosine	/	/	√	√	√	/	/	/	/	√	^51–53^
40	Populin	/	/	/	/	/	/	/	/	/	/	/
41	Pinoresinol 4-O-glucoside	/	/	/	/	/	/	/	/	/	/	/
42	6-O-Feruloylglucose	/	/	/	/	/	/	/	/	/	/	/
43	Pinostilbenoside	/	/	/	/	/	/	/	/	/	/	/
44	Desrhamnosylmartynoside	/	/	/	/	/	/	/	/	/	/	/
45	7-O-Methylaloeasinol	/	/	/	/	/	/	/	/	/	/	/
**Quinone compounds**												
46	4’-Hydroxypiptocarphin A	/	/	/	/	/	/	/	/	/	/	/
47	2-Hydroxy-3-methoxybenzoic acid glucose ester	/	/	√	/	/	/	/	/	/	/	^54^
48	2-Methoxy-1,4-naphthoquinone	/	√	√	√	√	√	/	/	/	/	^44,53,55–59,59,59–62,64,65,67^
**Others**												
49	Emodin-1-O-beta-gentiobioside	/	/	/	/	/	/	/	/	/	/	/
50	6’-(p-Hydroxybenzoyl) mussaenosidic acid	/	/	/	/	/	/	/	/	/	/	/
51	Loganate	/	/	/	/	/	/	/	/	/	/	/
52	Tetillapyrone	/	/	/	/	/	/	/	/	/	/	/
53	Granatomycin E	/	/	/	/	/	/	/	/	/	/	/

“√” means having this feature; “/” means not mentioned.

Table 4 summarizes the biological functions and potential applications of the newly identified compounds in BTL to provide a clearer understanding of its nutritional value. Among the 53 newly identified compounds in BTL, several exhibit diverse and significant biological activities, including antioxidant (9 compounds), antibacterial (7), anti-inflammatory (14), enzyme inhibition (6), anti-tumor (12), lipid-lowering (5), nutritional supplementation (3), anti-fatigue (2), blood pressure-lowering (1), and cardiovascular/cerebrovascular protective effects (2). Notably, Oleuropein aglycone, known for its potent antioxidant and anti-inflammatory activities^43,63^ and Vicenin II, a C-glycosyl flavonoid with demonstrated lipid-regulating and hepatoprotective effects, are among the most functionally versatile compounds^7,8^. In addition, 2-Methoxy-1,4-naphthoquinone and Isoschaftoside have been reported to exhibit strong anti-tumor and autophagy-activating properties, respectively^56,66^. Gly-Leu, a dipeptide with nutritional and bioactive potential^30,31^ and Adenosine, which plays key roles in energy metabolism and neuroprotection^51,52,67^ also contribute significantly to the enhanced health functionality of BTL. Following bamboo-tube brewing, BTL contains numerous additional bioactive compounds compared to BBL. These include 9 amino acids, 3 phenolic compounds, 8 terpenoids, 16 glycosides, 3 quinones, and 5 other compounds.

#### Flavonoids and isoflavonoids

Flavonoids and isoflavonoids are widely distributed in plants, and their basic skeleton contains two benzene rings (A and B rings) linked by a three-carbon bridge, often bearing multiple phenolic hydroxyl groups. Flavonoids and isoflavonoids exhibit a wide range of biological activities, and variations in their structures underlie their distinct functional properties^68^.

As shown in Table 4, after two months of sealed brewing, BTL contained six new flavonoids (Vicenin II, Vicenin III, Calealactone B, Epmedin C, Jacein and Yukovanol) and three isoflavonoids (Phomopsinone D, Isoschaftoside, and Norwogonin-8-O-glucuronide). These compounds originate from Moso bamboo tubes and gradually migrate into BBL after brewing. These flavonoids and Isoflavonoids exhibit various health-promoting biological activities, including antioxidant, antibacterial, anti-inflammatory, and anti-tumor properties^69^.

Notably, beyond the functions summarized in Table 4, some of these compounds possess additional bioactivities that are not discussed in the table. For example, Park et al. reported that Vicenin II inhibited hepatic lipid accumulation and reduced adipocyte size by suppressing lipogenesis. Vicenin II exhibited the strongest inhibitory effect on lipid accumulation in HepG2 cells co-cultured with differentiated 3T3-L1 and oleic acid (OA), among various glycosylated flavonoids. It significantly downregulated lipid metabolism–related genes, including FAS, PPARγ, FABP4, and SREBP1c^70^. In Sánchez’s study, Calealactone B had a gastric protective effect in an ethanol induced gastric injury model, and the possible involvement of prostaglandins, nitric oxide, and thiol groups in the mechanism of Calealactone B action was also evaluated^71^. Su et al. reported that Isoschaftoside may ameliorate non-alcoholic fatty liver disease (NAFLD) by activating autophagy, highlighting its potential clinical value in treating NAFLD^66^.

#### Amino acid

Amino acids are the fundamental units that constitute proteins and are crucial for the growth, development, and metabolic processes of living organisms^72,73^. Amino acids serve a variety of essential physiological functions. First, amino acids are utilized by the human body to synthesize antibodies that combat bacterial and viral infections^74^. Second, amino acids are involved in the synthesis of hemoglobin, which facilitates oxygen transport. Third, amino acids contribute to the production of enzymes and hormones that maintain metabolic balance^75^. Fourth, amino acids serve as essential building blocks for the formation of sperm and egg cells^76^. Lastly, amino acids are involved in the synthesis of neurotransmitters^77^.

Compared to BBL, BTL contains nine additional amino acids: D-Tryptophan, Glycyltyrosine, Ala-Glu-Leu, Gly-Leu, Asp-Ile-Phe, N-Acetyl tryptophan, Ser-Leu-Ile, Asp-Phe, and Ile-Leu-Glu^5,78^. As shown in Table 4, these amino acid compounds exhibit anti-tumor, antibacterial, antioxidant, anti-inflammatory, and nutritional functions. D-Tryptophan, Glycyltyrosine, Gly-Leu and N-acetyl tryptophan exhibit multiple biological effects. D-tryptophan has also been studied as an immune modulator, potentially exerting its effects by influencing immune cell function and modulating immune responses^79,80^.

In addition, some compounds exhibit additional activities beyond those summarized in Table 4. Gly-Leu exhibits radioprotective properties and facilitates protein transport^31,33^. N-acetyltryptophan has been shown to be beneficial in the treatment of polycystic ovary syndrome (PCOS)^36^.

#### Terpenoids

Terpenoids are a class of natural compounds composed of isoprene units, which are assembled through various linkage patterns. They are widely distributed in fruits, vegetables, medicinal plants, and whole grains^81^. Terpenoids can reduce cancer incidence, lower total and low-density lipoprotein (LDL) cholesterol levels, and decrease the risk of cardiovascular disease^82,83^.

As shown in Table 4, BTL contains eight newly identified terpenoid compounds compared to BBL, namely: Fibleucin, Morindin, Secologanic acid, Paeonilactone C, Oleuropein aglycone, (+)-Abscisic acid, Ciwujiatone and Zeylenol. Several of these compounds exhibit anti-inflammatory, antioxidant, and anti-tumor properties.

Beyond the effects summarized in Table 4, some compounds possess additional pharmacological activities. For example, Pratama et al. reported that Fibleucin may act as a neuraminidase inhibitor, suggesting its potential role in lipid metabolism modulation^84^. Oleuropein aglycone exhibits a broad spectrum of biological activities, including anti-inflammatory, antibacterial, and anti-tumor effects^85^; it also helps lower blood pressure, blood glucose, and cholesterol levels. Additionally, it has shown therapeutic potential in the treatment of Alzheimer’s disease^60^ and cardiovascular conditions^62^.

#### Glycoside compounds

Glycosides are formed by linking the anomeric carbon of a sugar or sugar derivative to a non-sugar moiety (aglycone) through a glycosidic bond. BTL contains 16 newly identified glycoside compounds, which may contribute to the increased sweetness of the liquor. The elevated levels of glycosides can enhance both the taste and aftertaste of the liquor^86^. Moreover, the pharmacological functions and development of glycoside compounds have garnered increasing attention in the field of traditional Chinese medicine^87^.

As shown in Table 4, sixteen glycoside compounds were newly identified in BTL. These include Torosachrysone 8-O-beta-gentiobioside, Forsythenside A, Sibiricose A5, 3-Deoxyguanosine, N2, N2-dimethylguanosine, Eugenol gentiobioside, Acanthoside B, Orcinol gentiobioside, Adenosine, Populin, Pinoresinol 4-O-glucoside, 6-O-Feruloylglucose, Pinostilbenoside, Desrhamnosylmartynoside, 7-O-Methylaloesinol and Fraxamoside. Several of these compounds exhibit anti-inflammatory, antioxidant, and anti-tumor properties.

Beyond the functions summarized in Table 4, several compounds demonstrate additional pharmacological effects. Sibiricose A5 demonstrates nootropic activity, contributing to improved mental performance^88,89^. Acanthoside B exhibits anti-inflammatory and anti-amnesic activities and has been extensively studied in the context of Alzheimer’s disease and pulmonary inflammation^90,91^. Adenosine plays important roles in energy metabolism, vasodilation, and neural regulation, and also serves as a therapeutic target in various pharmacological applications^51,92^. In Deng’s study, Forsythenside A effectively inhibited influenza A virus replication in mice, thereby improving disease prognosis^93^. In Niu’s study, Forsythenside A exhibited neuroprotective effects in MPTP-induced Parkinson’s disease mouse models^94^.

#### Quinone compounds

There are two common forms of quinone compounds: quinol and quinone. Quinone compounds, widely distributed in nature, can be obtained from plants, animals, and microorganisms^95^. Quinone compounds exhibit antioxidant, anti-tumor^96^ and antibacterial^97^ properties. They also participate in electron transfer, redox reactions, and molecular signal transduction, making them widely applicable in medicine and dye industries^98,99^.

As shown in Table S2, three new quinone compounds were identified in BTL: 4’-Hydroxypiptocarphin A, 2-Hydroxy-3-methoxybenzoic acid glucose ester, 2-Methoxy-1,4-naphthoquinone. Among these compounds, 2-Methoxy-1,4-naphthoquinone stands out for its broad-spectrum bioactivities, including antibacterial, anti-inflammatory, enzyme inhibitory, anti-tumor and lipid-lowering properties. Its multifunctionality significantly contributes to the enhanced health-promoting characteristics of BTL^56,64^.

Beyond the activities listed in Table 4, several compounds demonstrate additional biological effects. For instance, Huh’s study reported that 2-Hydroxy-3-methoxybenzoic acid glucose ester exhibited effective antagonistic activity against platelet-activating factor (PAF). This suggests its potential protective role in treating peripheral circulatory disorders and PAF-induced allergic shock^100^. 2-Methoxy-1,4-naphthoquinone has also been shown to induce apoptosis in A549 lung cancer cells via activation of the JNK and p38 MAPK signaling pathways^56^.

In summary, most of the 53 newly identified compounds in BTL exhibit various beneficial biological effects, supporting the conclusion that BTL is highly nutritious and providing a scientific basis for its health-promoting potential.

#### Changes in chemical composition content

**Fig. 4:**
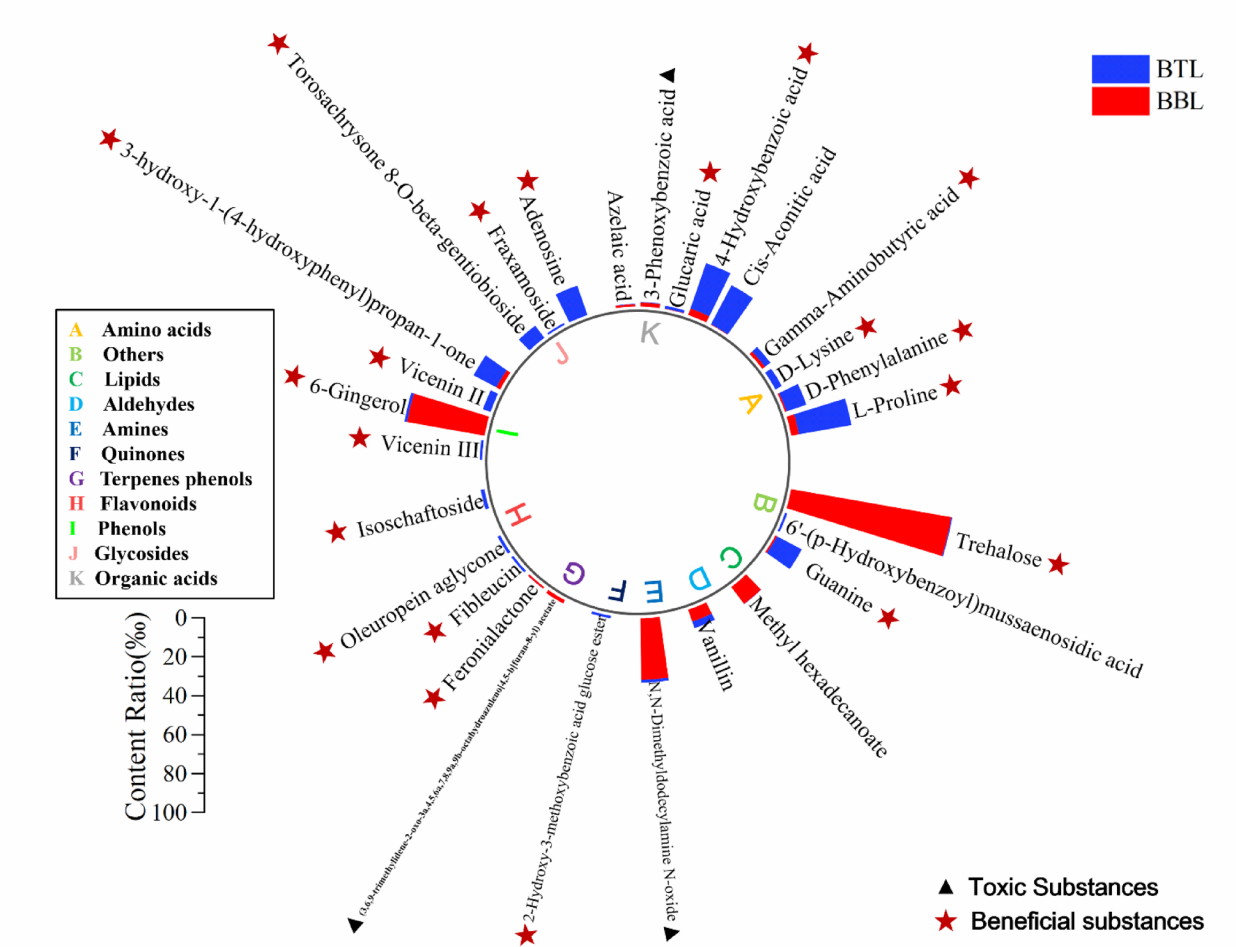
Changes in compound content ratio between BBL and BTL (variation amplitude ≥ 1‰, Fragmentation Score ≥ 80).

After being sealed and brewed in fresh bamboo tubes for two months, the chemical composition of BBL changed. To assess whether these changes are beneficial or detrimental to human health, compounds with a fragmentation score ≥ 80 and a relative content change ≥ 1‰ were selected for comparative analysis. A total of 28 compounds were selected from BBL, and the results are summarized in Fig. 4. As shown in Fig. 4, there was a significant increase in the content of four amino acids, three organic acids, three glycosides, and two phenolic compounds, all of which are beneficial to the human body. For example, L-Proline and 4-hydroxybenzoic acid are physiologically important compounds for the human body^101,102^. Phenolic compounds such as 3-hydroxy-1-(4-hydroxyphenyl)propan-1-one and Vicenin II exhibit antioxidant and anti-inflammatory activities^13,103^. The three glycosides with increased content are also known for their antioxidant or vasodilatory properties. Notably, the levels of three harmful compounds in BBL significantly decreased, particularly N, N-Dimethyldodecylamine N-oxide. This compound is a surfactant commonly used as a foam enhancer in detergents and is known to be harmful to human health^104^. The other two compounds—(3,6,9-trimethylidene-2-oxo-3a,4,5,6a,7,8,9a,9b-octahydroazuleno[4,5-b]furan-8-yl) acetate and 3-Phenoxybenzoic acid—are used in treating skin diseases and as pharmaceutical intermediates, respectively, and are not intended for human consumption^105^. In addition, compared with BBL, the relative content of 19 beneficial substances in BTL significantly increased, while the content of 3 harmful substances significantly decreased. It can be observed that brewing BBL in fresh Moso bamboo tubes not only reduces harmful compounds such as fusel alcohols and biogenic amines—likely through absorption and adsorption by bamboo-derived lignocellulosic components—but also enriches the liquor with beneficial bioactive compounds including flavonoids, glycosides, alkaloids, and polysaccharides that gradually migrate from the bamboo matrix into the BBL during storage.

## Experimental section

### Main materials

Moso bamboo tube: A 1-year-old bamboo cut from Yanling, Hunan province, with bamboo joints at both ends, and the segment taken was 1.5 m from bottom to top.

Beeswax: Purchased from Guangdong Southern BASF Wax Industry Co., Ltd.

Bitter buckwheat liquor (BBL): Purchased from Lanping Jinli Liquor Industry Co., Ltd., with an alcohol content of 42% vol; Main components of BBL: water, Tartary buckwheat.

### Preparation process of bamboo tube liquor (BTL)

**Fig. 5:**
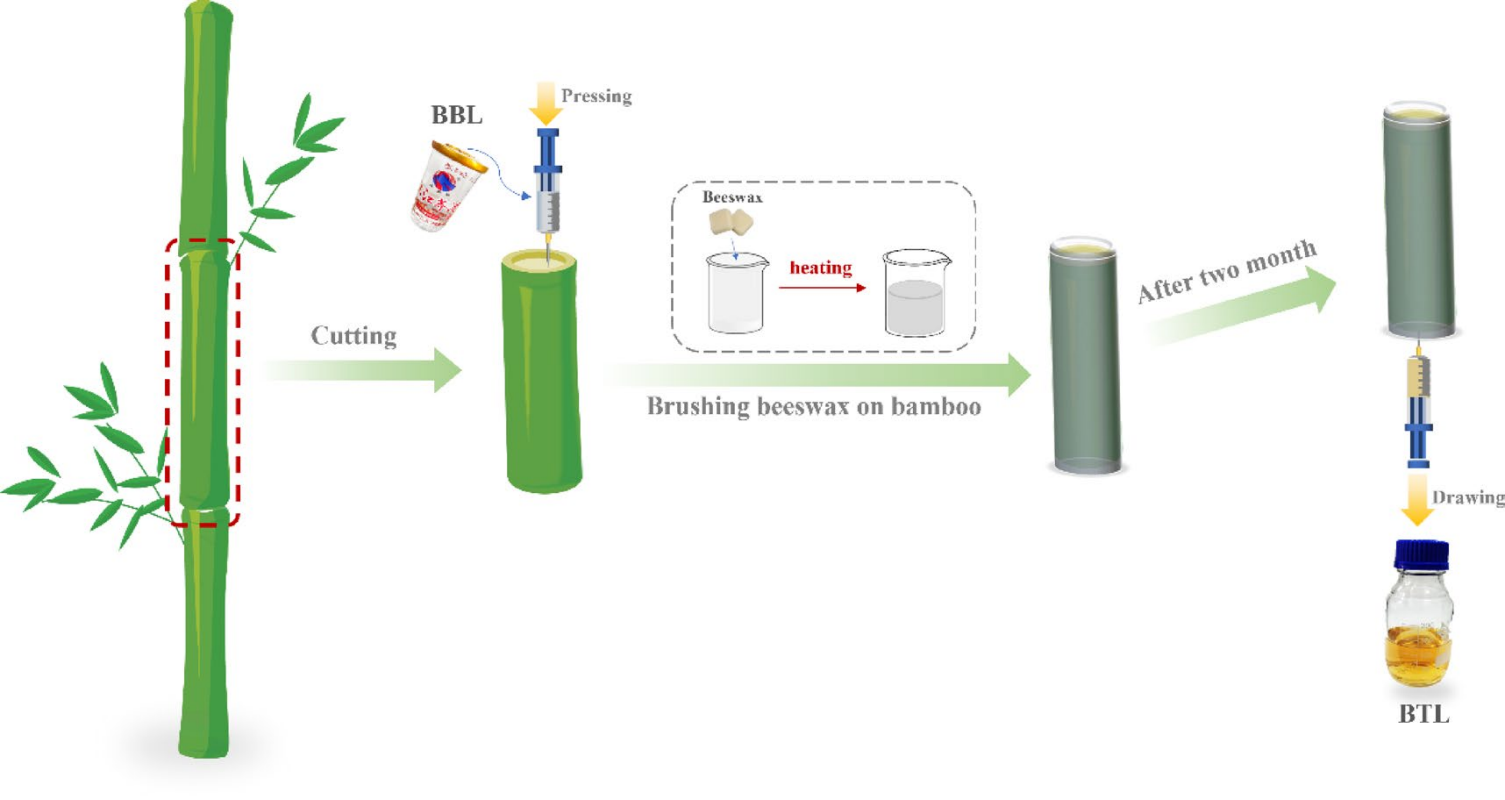
BTL Brewing Schematic.

As is shown in Fig. 5, inject 500 mL of sterilized BBL into freshly harvested Moso bamboo tubes that are one year old. After injection, evenly apply melted beeswax to the exterior of the Moso bamboo tubes. After being stored for two months, the BTL was extracted from the Moso bamboo tubes using a sterilized syringe. Store the BTL in a glass bottle at room temperature, away from light.

To ensure consistency and reproducibility, the sample preparation process was repeated three times to obtain three independent batches of samples. All measurements and characterizations were performed on each batch, and the results exhibited good agreement with minimal variation. For clarity and consistency in data presentation, the dataset from the batch with values closest to the average was selected for analysis and reporting in the main text.

### Alcohol content test

The ethanol concentration in the samples was determined following the guidelines of the National Food Safety Standard GB 5009.225–2016.

### Color determination

The CIELab colorimetric method was employed for analysis following the OIV harmonised guidelines for single laboratory validation (OIV-MA-AS1-13, 2008)^106^. Specifically, 3 mL of the sample were taken, passed through a 0.22 μm organic filter membrane, and 2 mL of the sample were taken in a colorimetric dish. Absorbance values were measured at wavelengths of 440, 530, and 600 nm using a UV-visible spectrophotometer to calculate the L^*^, a^*^, and b^*^ values.

### Odor testing of volatile gases

The PEN3 model from AIRSENSE, Germany, was used to simulate the human olfactory system for odor assessment. 18 mL sample was taken in a 40 mL headspace vial with a polytetrafluoroethylene cap, and detection was performed at room temperature.

### Total sugar content testing

Total sugar content was measured using the direct titration method according to GB/T 15,038 − 2006.

### Total flavonoid testing

Flavonoid content was determined according to NY/T 1295–2007, a standard method for buckwheat and its products. Absorbance was measured with a UV spectrophotometer.

### Total phenol testing

Total phenolic content was measured according to T/NAIA 097-2021 using a UV spectrophotometer.

### FTIR (Fourier transform infrared Spectroscopy)

Fourier Transform Infrared Spectroscopy (FTIR) was performed using a Nicolet 6700 instrument (Thermo Scientific, USA) to analyze the chemical functional groups within the sample in the 4000–500 cm⁻¹ range.

### LC-MS (Liquid Chromatography-Mass Spectrometry)

A 500 µL aliquot of each sample was centrifuged at 12,000 *g* for 10 min at 4 °C. The resulting supernatant was collected and filtered through a 0.22 μm membrane filter before being subjected to instrumental analysis^107^.

#### UPLC conditions

Chromatographic separation was performed using a Waters HSS T3 column (100 × 2.1 mm, 1.8 μm). The mobile phases were: solvent A, 0.1% formic acid in water; and solvent B, 0.1% formic acid in acetonitrile. The flow rate was set at 0.3 mL/min, the column temperature was maintained at 40 °C, and the injection volume was 2 µL. The elution gradient was as follows: 0.0–1.0 min, 0% B; 1.0–9.0 min, linear increase from 0 to 95% B; 9.0–13.0 min, 95% B; 13.0–13.1 min, linear decrease from 95 to 0% B; 13.1–17.0 min, 0% B. Throughout the analysis, samples were stored in a 4 °C autosampler. To minimize the impact of instrument signal fluctuations, samples were analyzed in a randomized order. Quality control (QC) samples were inserted throughout the sample queue to monitor system stability and ensure data reliability.

#### Mass spectrometry conditions

Mass spectrometric data were acquired using the Q Exactive HFX high-resolution mass spectrometer (Thermo, USA) operating in both positive and negative electrospray ionization (ESI) modes. The ESI conditions were as follows: sheath gas flow rate, 40 arbitrary units (arb); auxiliary gas flow rate, 10 arb; spray voltage, + 3000 V/–2800 V; capillary temperature, 350 °C; and ion transfer tube temperature, 320 °C. Data were collected in Full MS/ddMS² mode. The mass scan range was m/z 70–1050, with a resolution of 70,000 for MS¹ and 17,500 for MS².

#### Data analysis and interpretation

**Fig. 6:**
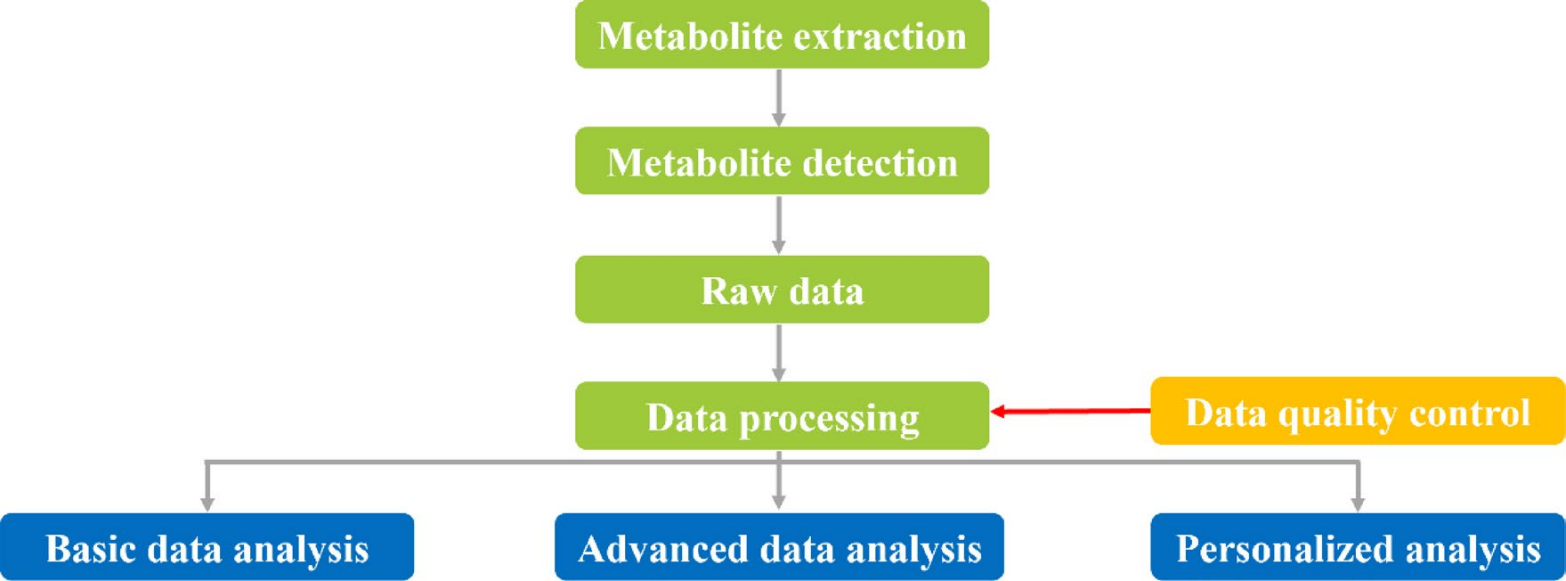
LC-MS Analysis Procedure.

The LC-MS analysis process is shown in Fig. 6. Raw data were processed using the Progenesis QI metabolomics processing software (Waters Corporation, Milford, USA) for baseline filtering, peak recognition, integration, retention time correction, and peak alignment, ultimately resulting in a data matrix of retention time, mass-to-charge ratio, and peak intensity. Primary databases used included http://www.hmdb.ca/, https://metlin.scripps.edu/, and other public databases, as well as a self-built database. Substances with fragmentation scores of 80 or higher were selected as analytical components.

## Supplementary Information

Below is the link to the electronic supplementary material.

Supplementary Material 1

## Data Availability

The original contributions presented in this study are included in the article. Further inquiries can be directed to the corresponding author.
