# Supplementary material for: Effects of Moso bamboo tubes on color, aroma, physicochemical properties and composition of bitter buckwheat liquor
Source: Sci Rep. 2025 Aug 27;15:31608. doi: 10.1038/s41598-025-16966-7 (PMC12391343; doi:10.1038/s41598-025-16966-7)
Supplement: Supplementary file 1 — Supplementary Material 1 [file 41598_2025_16966_MOESM1_ESM.docx]

**Supplementary Material**

**Table S1 LCMS analysis data of BBL and TBL**

| **No.** | **Metabolite** | **Fragmentation Score** | **Retention time(min)** | **Formula** | **m/z** | **Content ratio in BBL（%）** | **Content ratio in TBL（%）** |
| --- | --- | --- | --- | --- | --- | --- | --- |
| _1_ | (-)-Syringaresinol di-O-glucoside | 98 | 5.23390 | C_34_H_46_O_18_ | 741.2624597 | 0.000000000 | 0.000533070 |
| _2_ | Syringic acid | 97.4 | 4.87367 | C_9_H_10_O_5_ | 197.0450549 | 0.000399033 | 0.000928802 |
| _3_ | 4-Hydroxybenzoic acid | 97.1 | 6.16908 | C_7_H_6_O_3_ | 137.0233822 | 0.003505066 | 0.024629742 |
| _4_ | D-phenylalanine | 96.8 | 1.97848 | C_9_H_11_NO_2_ | 164.0708964 | 0.000839327 | 0.010761781 |
| _5_ | D-Lysine | 96.2 | 0.66593 | C_6_H_14_N_2_O_2_ | 147.1128385 | 0.000030315 | 0.003697418 |
| _6_ | Vicenin II | 96.1 | 4.95020 | C_27_H_30_O_15_ | 593.1519451 | 0.000000000 | 0.004129280 |
| _7_ | trehalose | 96 | 0.85005 | C_12_H_22_O_11_ | 377.0856687 | 0.083660771 | 0.000448365 |
| _8_ | Sinapinic acid | 95.5 | 5.52003 | C_11_H_12_O_5_ | 223.0608938 | 0.000278103 | 0.001081919 |
| _9_ | D-Tryptophan | 94.5 | 3.86757 | C_11_H_12_N_2_O_2_ | 203.0821295 | 0.000006487 | 0.000596064 |
| _10_ | Guanine | 94.5 | 0.97668 | C_5_H_5_N_5_O | 152.0567308 | 0.000968441 | 0.014424529 |
| _11_ | Vicenin III | 94.5 | 5.31200 | C_26_H_28_O_14_ | 565.1554578 | 0.000000000 | 0.001258012 |
| _12_ | L-Tyrosine | 93.6 | 1.14903 | C_9_H_11_NO_3_ | 180.0659415 | 0.000207868 | 0.001151346 |
| _13_ | Methyl chlorogenate | 93.6 | 5.27270 | C_17_H_20_O_9_ | 367.103683 | 0.000047764 | 0.000792158 |
| _14_ | Coniferaldehyde | 93.5 | 6.69218 | C_10_H_10_O_3_ | 177.0551018 | 0.000193460 | 0.000120588 |
| _15_ | Isoschaftoside | 93.5 | 5.32323 | C_26_H_28_O_14_ | 563.1412446 | 0.000028564 | 0.001875004 |
| _16_ | L-Proline | 93.4 | 0.82392 | C_5_H_9_NO_2_ | 116.0709713 | 0.004010815 | 0.027386933 |
| _17_ | Glycyltyrosine | 93.3 | 1.07430 | C_11_H_14_N_2_O_4_ | 239.1025676 | 0.000000000 | 0.000363904 |
| _18_ | Neoschaftoside | 92.8 | 5.20748 | C_26_H_28_O_14_ | 563.1411049 | 0.000855788 | 0.001378840 |
| _19_ | Sibiricose A5 | 92.5 | 4.73443 | C_22_H_30_O_14_ | 517.1565335 | 0.000000000 | 0.000677916 |
| _20_ | Vanillic acid | 92.2 | 5.68820 | C_8_H_8_O_4_ | 167.0342399 | 0.000662889 | 0.000076168 |
| _21_ | Glucaric acid | 92.1 | 0.82923 | C_6_H_10_O_8_ | 209.0297805 | 0.000164458 | 0.001500666 |
| _22_ | Emodin-1-O-beta-gentiobioside | 91.7 | 5.52003 | C_27_H_30_O_15_ | 575.1410392 | 0.000000000 | 0.000690858 |
| _23_ | Morindin | 90.9 | 5.58412 | C_26_H_28_O_14_ | 545.1305171 | 0.000000000 | 0.000335537 |
| _24_ | 9-(2,3-Dihydroxypropoxy)-9-oxononanoic acid | 90.6 | 6.14263 | C_12_H_22_O_6_ | 261.1343097 | 0.000653956 | 0.000078201 |
| _25_ | Forsythenside A | 90.4 | 7.22663 | C_22_H_26_O_10_ | 451.1599881 | 0.000000000 | 0.000150087 |
| _26_ | Epmedin C | 90 | 6.11628 | C_39_H_50_O_19_ | 821.2889567 | 0.000000000 | 0.000979340 |
| _27_ | Laminine | 89.9 | 0.72083 | C_9_H_20_N_2_O_2_ | 189.1599508 | 0.000025793 | 0.000106348 |
| _28_ | gamma-Aminobutyric acid | 89.7 | 0.78347 | C_4_H_9_NO_2_ | 104.0711188 | 0.001812977 | 0.003484948 |
| _29_ | 6'-(p-Hydroxybenzoyl)mussaenosidic acid | 89.3 | 5.18110 | C_23_H_28_O_12_ | 495.1512755 | 0.000000000 | 0.001141464 |
| _30_ | Fraxamoside | 89.3 | 5.22100 | C_25_H_30_O_13_ | 583.1675421 | 0.000000000 | 0.001019203 |
| _31_ | 2-methoxy-4-[3-[3-(trifluoromethyl)anilino]imidazo[1,2-a]pyrimidin-2-yl]phenol | 89 | 6.47733 | C_20_H_15_F_3_N_4_O_2_ | 401.1209819 | 0.000000000 | 0.000105117 |
| _32_ | 3-hydroxy-1-(4-hydroxyphenyl)propan-1-one | 88.8 | 4.82342 | C_9_H_10_O_3_ | 165.0548976 | 0.002311663 | 0.013245349 |
| _33_ | Methyl hexadecanoate | 88.8 | 10.82835 | C_17_H_34_O_2_ | 315.2540844 | 0.010952740 | 0.000134824 |
| _34_ | Pyridoxine | 88.7 | 0.98767 | C_8_H_11_NO_3_ | 170.0812403 | 0.000204432 | 0.000308086 |
| _35_ | 9-Oxo-12,13-Epoxy-10-Octadecenoic acid | 88.6 | 9.60245 | C_18_H_30_O_4_ | 275.2004983 | 0.000073486 | 0.000560696 |
| _36_ | Adenosine | 88.1 | 1.64648 | C_10_H_13_N_5_O_4_ | 268.1038863 | 0.000000000 | 0.015934154 |
| _37_ | Ciwujiatone | 88.1 | 8.07605 | C_44_H_50_O_18_ | 867.3070319 | 0.000000000 | 0.000993270 |
| _38_ | Guanosine | 88.1 | 1.36173 | C_10_H_13_N_5_O_5_ | 282.0843292 | 0.000010092 | 0.000566390 |
| _39_ | Vanillin | 88.1 | 5.89728 | C_8_H_8_O_3_ | 151.0392244 | 0.006442446 | 0.003548170 |
| _40_ | Jaceosidin | 88 | 7.93020 | C_17_H_14_O_7_ | 329.0668073 | 0.000057445 | 0.000236744 |
| _41_ | 4'-Hydroxypiptocarphin A | 87.7 | 5.92250 | C_21_H_26_O_10_ | 459.1284171 | 0.000000000 | 0.000103884 |
| _42_ | Feronialactone | 87.7 | 10.49813 | C_19_H_22_O_3_ | 343.1550994 | 0.001086230 | 0.000028994 |
| _43_ | Corymboside | 87.6 | 5.19033 | C_26_H_28_O_14_ | 565.1554554 | 0.000037041 | 0.000820950 |
| _44_ | Ala Glu Leu | 87.4 | 4.00308 | C_14_H_25_N_3_O_6_ | 332.1815662 | 0.000000000 | 0.000285499 |
| _45_ | Fibleucin | 86.8 | 7.52072 | C_20_H_20_O_6_ | 357.1330872 | 0.000000000 | 0.001053806 |
| _46_ | Ethyl ferulate | 86.7 | 4.64395 | C_12_H_14_O_4_ | 221.0815633 | 0.000217858 | 0.000164565 |
| _47_ | Gly Leu | 86.7 | 1.99107 | C_8_H_16_N_2_O_3_ | 187.1082071 | 0.000000000 | 0.000259689 |
| _48_ | Monoethyl phthalate | 86.6 | 9.62753 | C_10_H_10_O_4_ | 177.0546433 | 0.000702746 | 0.000044913 |
| _49_ | 9-Hydroxy-10,12-octadecadienoic acid | 86.3 | 10.81463 | C_18_H_32_O_4_ | 311.2228257 | 0.000732038 | 0.000287349 |
| _50_ | Asp Ile Phe | 86.3 | 5.73440 | C_19_H_27_N_3_O_6_ | 394.197297 | 0.000000000 | 0.000116068 |
| _51_ | Chromomoric acid B | 86.3 | 10.53595 | C_18_H_28_O_3_ | 293.2109893 | 0.000353675 | 0.000041330 |
| _52_ | N,N-Dimethyldodecylamine N-oxide | 86.3 | 8.77773 | C_14_H_31_NO | 230.2478806 | 0.031652904 | 0.001468785 |
| _53_ | (3,6,9-trimethylidene-2-oxo-3a,4,5,6a,7,8,9a,9b-octahydroazuleno[4,5-b]furan-8-yl) acetate | 86.2 | 11.01642 | C_17_H_20_O_4_ | 352.1527117 | 0.001987589 | 0.000019345 |
| _54_ | N-Acetyl-tryptophan | 86.2 | 5.83210 | C_13_H_14_N_2_O_3_ | 245.0930644 | 0.000000000 | 0.000131609 |
| _55_ | Pinoresinol 4-O-glucoside | 86.2 | 6.41438 | C_26_H_32_O_11_ | 519.1877603 | 0.000000000 | 0.000262747 |
| _56_ | Azelaic acid | 86.1 | 8.24380 | C_9_H_16_O_4_ | 169.0863103 | 0.001239686 | 0.000157978 |
| _57_ | 6-Gingerol | 85.9 | 9.53505 | C_17_H_26_O_4_ | 293.1759076 | 0.040415723 | 0.001314088 |
| _58_ | Inosine | 85.8 | 6.55158 | C_9_H_7_NO | 146.0600679 | 0.000137418 | 0.000213497 |
| _59_ | Phomopsinone D | 85.8 | 4.27118 | C_12_H_16_O_5_ | 273.1331572 | 0.000000000 | 0.000393396 |
| _60_ | trans-2,3-Dihydro-3-hydroxyeuparin | 85.7 | 6.10400 | C_13_H_14_O_4_ | 279.0874392 | 0.000185871 | 0.000118159 |
| _61_ | Populin | 85.5 | 7.17878 | C_20_H_22_O_8_ | 373.1281861 | 0.000000000 | 0.000091076 |
| _62_ | Granatomycin E | 85.3 | 5.02835 | C_22_H_22_O_11_ | 507.1148681 | 0.000000000 | 0.000289808 |
| _63_ | Ile Leu Glu | 85.3 | 4.11945 | C_17_H_31_N_3_O_6_ | 374.2284579 | 0.000000000 | 0.000224947 |
| _64_ | Brosimacutin G | 85.1 | 7.57893 | C_20_H_20_O_6_ | 401.1241858 | 0.000015228 | 0.000179701 |
| _65_ | Torosachrysone 8-O-beta-gentiobioside | 85.1 | 5.12920 | C_28_H_36_O_15_ | 611.1988792 | 0.000000000 | 0.007827903 |
| _66_ | 6-O-Feruloylglucose | 84.8 | 2.31387 | C_16_H_20_O_9_ | 401.1090681 | 0.000000000 | 0.000132714 |
| _67_ | 7-O-Methylaloeasinol | 84.5 | 6.41752 | C_20_H_26_O_9_ | 375.1435946 | 0.000000000 | 0.000154367 |
| _68_ | cis-Aconitic acid | 84.2 | 1.03813 | C_6_H_6_O_6_ | 173.0084011 | 0.000342331 | 0.022600358 |
| _69_ | Secologanic acid | 83.9 | 1.20513 | C_16_H_22_O_10_ | 419.1197001 | 0.000000000 | 0.000662947 |
| _70_ | Methyl 4-hydroxycinnamate | 83.8 | 1.00782 | C_10_H_10_O_3_ | 179.0703064 | 0.000793723 | 0.000279274 |
| _71_ | Paeonilactone C | 83.8 | 5.55717 | C_17_H_18_O_6_ | 363.1087108 | 0.000000000 | 0.000198960 |
| _72_ | Ser Leu Ile | 83.8 | 5.15377 | C_15_H_29_N_3_O_5_ | 332.2179063 | 0.000000000 | 0.000131268 |
| _73_ | Zeylenol | 83.6 | 6.46408 | C_21_H_20_O_7_ | 417.1543175 | 0.000000000 | 0.000895985 |
| _74_ | 3-Deoxyguanosine | 83.5 | 1.48558 | C_10_H_13_N_5_O_4_ | 266.0895188 | 0.000000000 | 0.000864258 |
| _75_ | Yukovanol | 83.5 | 6.55158 | C_20_H_18_O_6_ | 387.1436386 | 0.000000000 | 0.000167000 |
| _76_ | 2-[(1S,2S,4aR,8aS)-1-hydroxy-4a-methyl-8-methylidene-1,2,3,4,5,6,7,8a-octahydronaphthalen-2-yl]prop-2-enoic acid | 83.2 | 12.33422 | C_15_H_22_O_3_ | 249.1495063 | 0.000664708 | 0.000016610 |
| _77_ | Ethyl syringate | 83.1 | 7.82557 | C_11_H_14_O_5_ | 225.0765636 | 0.000145584 | 0.000126538 |
| _78_ | Asp Phe | 82.9 | 4.09692 | C_13_H_16_N_2_O_5_ | 281.1131559 | 0.000000000 | 0.000438499 |
| _79_ | Eugenol gentiobioside | 82.9 | 4.89858 | C_22_H_32_O_12_ | 509.1648423 | 0.000000000 | 0.000167508 |
| _80_ | Pinostilbenoside | 82.7 | 7.86507 | C_21_H_24_O_8_ | 807.2878662 | 0.000000000 | 0.000267573 |
| _81_ | Jacein | 82.5 | 5.04040 | C_24_H_26_O_13_ | 567.1361027 | 0.000000000 | 0.000466031 |
| _82_ | Tetillapyrone | 82.4 | 4.17935 | C_11_H_14_O_6_ | 284.112756 | 0.000000000 | 0.000825289 |
| _83_ | coumarin | 82.1 | 5.98802 | C_9_H_6_O_2_ | 191.0344656 | 0.000133320 | 0.000139421 |
| _84_ | Loganate | 82.1 | 4.81058 | C_16_H_24_O_10_ | 357.1192412 | 0.000000000 | 0.000155703 |
| _85_ | Securiterpenoside | 82.1 | 4.29472 | C_11_H_18_O_8_ | 243.0863316 | 0.000025146 | 0.000706675 |
| _86_ | 2-Methoxy-1,4-naphthoquinone | 82 | 5.57060 | C_11_H_8_O_3_ | 187.0395518 | 0.000000000 | 0.000339462 |
| _87_ | Norwogonin-8-O-glucuronide | 81.7 | 5.40242 | C_21_H_18_O_11_ | 445.0780936 | 0.000000000 | 0.000273687 |
| _88_ | Desrhamnosylmartynoside | 81.5 | 6.29700 | C_25_H_30_O_11_ | 507.1862464 | 0.000000000 | 0.000146691 |
| _89_ | (+)-Abscisic acid | 81.2 | 7.18863 | C_15_H_20_O_4_ | 263.1288648 | 0.000000000 | 0.000046565 |
| _90_ | Indole-3-carboxylic acid | 81.1 | 6.38688 | C_9_H_7_NO_2_ | 160.0396368 | 0.000057976 | 0.000062935 |
| _91_ | 2-Hydroxy-3-methoxybenzoic acid glucose ester | 81 | 6.16125 | C_14_H_18_O_9_ | 295.0812643 | 0.000000000 | 0.001192451 |
| _92_ | 2-Methoxy-5-nitrophenol | 81 | 7.30578 | C_7_H_7_NO_4_ | 168.0295412 | 0.000120371 | 0.000076097 |
| _93_ | Calealactone B | 80.9 | 6.65315 | C_21_H_26_O_9_ | 403.1399597 | 0.000000000 | 0.000547189 |
| _94_ | 3-Phenoxybenzoic acid | 80.8 | 9.33852 | C_13_H_10_O_3_ | 213.0553524 | 0.002029438 | 0.000293951 |
| _95_ | 9-Oxo-10E,12Z-octadecadienoic acid | 80.8 | 11.95347 | C_18_H_30_O_3_ | 295.226834 | 0.000252439 | 0.000009771 |
| _96_ | 13-Hydroperoxy-(9Z,11E)-octadecadienoic acid | 80.7 | 10.13407 | C_18_H_32_O_4_ | 311.2229144 | 0.000380320 | 0.000897437 |
| _97_ | 15,16-Dihydroxyoctadeca-9Z,12Z-dienoic acid | 80.6 | 10.03445 | C_18_H_32_O_4_ | 295.2267476 | 0.000340915 | 0.000909905 |
| _98_ | 9-O-Feruloyl-5,5'-dimethoxylariciresinol | 80.5 | 7.34353 | C_32_H_36_O_11_ | 641.2247633 | 0.000000000 | 0.000173267 |
| _99_ | N2,N2-Dimethylguanosine | 80.5 | 3.71282 | C_12_H_17_N_5_O_5_ | 310.1157487 | 0.000000000 | 0.000077910 |
| _100_ | Acanthoside B | 80.2 | 5.23390 | C_28_H_36_O_13_ | 579.2090512 | 0.000000000 | 0.000549059 |
| _101_ | Orcinol gentiobioside | 80.2 | 1.43515 | C_19_H_28_O_12_ | 493.1569228 | 0.000000000 | 0.000329004 |
| _102_ | 4-Hydroxycinnamic acid | 80 | 5.09313 | C_9_H_8_O_3_ | 147.0441476 | 0.000040492 | 0.000189022 |
| _103_ | Oleuropein aglycone | 80 | 5.92250 | C_19_H_22_O_8_ | 377.1243539 | 0.000000000 | 0.001591076 |
| _104_ | Tanshindiol A | 79.8 | 6.90127 | C_18_H_16_O_5_ | 311.0925584 | 0.000000000 | 0.000054773 |
| _105_ | 6-O-Vanilloylajugol | 79.7 | 5.44275 | C_23_H_30_O_12_ | 479.1563079 | 0.000000000 | 0.000200021 |
| _106_ | Adenine | 79.7 | 2.93387 | C_5_H_5_N_5_ | 136.0618726 | 0.000165189 | 0.000204993 |
| _107_ | Glu Phe | 79.6 | 3.95687 | C_14_H_18_N_2_O_5_ | 295.128813 | 0.000000000 | 0.000433329 |
| _108_ | Securinine | 79.6 | 8.34882 | C_13_H_15_NO_2_ | 262.1084587 | 0.000000000 | 0.000031387 |
| _109_ | Massoniresinol | 79.3 | 5.23390 | C_20_H_24_O_8_ | 437.1455373 | 0.000000000 | 0.001604687 |
| _110_ | Chrysin-7-O-glucuronide | 79.1 | 5.19482 | C_21_H_18_O_10_ | 475.0885361 | 0.000000000 | 0.001100026 |
| _111_ | Salidroside | 79.1 | 5.22787 | C_14_H_20_O_7_ | 265.1069596 | 0.000000000 | 0.000171583 |
| _112_ | 2'-O-Methyladenosine | 78.8 | 2.93387 | C_11_H_15_N_5_O_4_ | 282.1195572 | 0.000000000 | 0.001567794 |
| _113_ | Salicortin | 78.8 | 6.78062 | C_20_H_24_O_10_ | 389.1229889 | 0.000000000 | 0.000229781 |
| _114_ | Epimedin C | 78.6 | 6.61312 | C_39_H_50_O_19_ | 803.2783155 | 0.000000000 | 0.000222154 |
| _115_ | Isorhapontin | 78.6 | 5.06632 | C_21_H_24_O_9_ | 465.1404238 | 0.000000000 | 0.000376988 |
| _116_ | Resveratroloside | 78.5 | 5.96468 | C_20_H_22_O_8_ | 391.1386 | 0.000000000 | 0.000112984 |
| _117_ | 1-O-(3-Hydroxy-4,5-dimethoxybenzoyl)hexopyranose | 78.4 | 3.63745 | C_15_H_20_O_10_ | 359.0985124 | 0.000000000 | 0.005240286 |
| _118_ | Phyllanthurinolactone | 78.4 | 4.93903 | C_14_H_18_O_8_ | 315.107413 | 0.000000000 | 0.000566172 |
| _119_ | Populoside | 78.4 | 5.59715 | C_22_H_24_O_10_ | 493.1355481 | 0.000000000 | 0.000526481 |
| _120_ | Glu Glu Ile | 78.2 | 4.11945 | C_16_H_27_N_3_O_8_ | 390.1871002 | 0.000000000 | 0.000162944 |
| _121_ | 13-Oxo-9,11-octadecadienoic acid | 78.1 | 11.73918 | C_18_H_30_O_3_ | 295.2267353 | 0.000186858 | 0.000051674 |
| _122_ | Methoxyeugenol 4-O-rutinoside | 78.1 | 6.15548 | C_23_H_34_O_12_ | 483.1876315 | 0.000000000 | 0.000091085 |
| _123_ | Orcinol 1-O-beta-D-apiofuranosyl-(1->6)-beta-D-glucopyranoside | 78.1 | 4.23568 | C_18_H_26_O_11_ | 463.1461701 | 0.000000000 | 0.000298499 |
| _124_ | Hydroxysafflor yellow A | 77.9 | 5.50932 | C_27_H_32_O_16_ | 577.1552163 | 0.000000000 | 0.000339412 |
| _125_ | Ile Val Gly | 77.8 | 1.91797 | C_13_H_25_N_3_O_4_ | 288.1915725 | 0.000025893 | 0.000181847 |
| _126_ | Violanthin | 77.8 | 5.52002 | C_27_H_30_O_14_ | 579.1710796 | 0.000000000 | 0.000659087 |
| _127_ | 4-Pyridoxic acid | 77.7 | 1.53588 | C_8_H_9_NO_4_ | 184.0605575 | 0.000000000 | 0.000257692 |
| _128_ | Coleonol B | 77.4 | 9.74467 | C_22_H_34_O_7_ | 391.2106416 | 0.000000000 | 0.000056949 |
| _129_ | P-Anisic acid | 77.4 | 4.39333 | C_8_H_8_O_3_ | 151.0392376 | 0.008894358 | 0.001661023 |
| _130_ | Asp Tyr | 77.3 | 1.64648 | C_13_H_16_N_2_O_6_ | 297.108107 | 0.000000000 | 0.000133424 |
| _131_ | Przewaquinone C | 77.3 | 7.42283 | C_18_H_16_O_4_ | 295.0974287 | 0.000011267 | 0.000069843 |
| _132_ | Sphinganine | 77.3 | 9.78652 | C_18_H_39_NO_2_ | 302.3052786 | 0.039418400 | 0.001115317 |
| _133_ | tubuloside B | 77.3 | 6.54675 | C_31_H_38_O_16_ | 647.1994029 | 0.000000000 | 0.000104408 |
| _134_ | Moracin P | 77.2 | 5.97428 | C_19_H_18_O_5_ | 371.1136863 | 0.000000000 | 0.000103624 |
| _135_ | Stearidonic acid | 77.2 | 12.02340 | C_18_H_28_O_2_ | 321.20711 | 0.001249780 | 0.000059704 |
| _136_ | L-HISTIDINE | 77 | 0.72897 | C_6_H_9_N_3_O_2_ | 154.0613697 | 0.000007119 | 0.000019853 |
| _137_ | Ligusticumic acid-methylester | 76.9 | 11.96578 | C_13_H_16_O_3_ | 262.143843 | 0.000400869 | 0.000026830 |
| _138_ | Rehmapicrogenin | 76.9 | 6.03872 | C_10_H_16_O_3_ | 551.3233096 | 0.000000000 | 0.000385148 |
| _139_ | Eriodictiol-7-glucoside | 76.8 | 5.52003 | C_22_H_24_O_11_ | 509.1303609 | 0.000000000 | 0.000312261 |
| _140_ | Chavibetol | 76.7 | 6.96165 | C_20_H_22_O_7_ | 339.1226226 | 0.000000000 | 0.000077533 |
| _141_ | p-Vinylphenyl O-[beta-D-apiofuranosyl-(1-6)]-beta-D-glucopyranoside | 76.7 | 5.90315 | C_19_H_26_O_10_ | 379.138676 | 0.000000000 | 0.000681345 |
| _142_ | (E)-Cinnamic acid | 76.6 | 6.44060 | C_9_H_8_O_2_ | 149.0597854 | 0.000206270 | 0.000328407 |
| _143_ | Gerberinside | 76.6 | 6.48967 | C_16_H_18_O_8_ | 339.1074803 | 0.000000000 | 0.000506682 |
| _144_ | N-Methyl-a-aminoisobutyric acid | 76.6 | 0.80363 | C_5_H_11_NO_2_ | 118.0864395 | 0.047164093 | 0.178523667 |
| _145_ | Curvulin | 76.5 | 6.00025 | C_12_H_14_O_5_ | 521.1668898 | 0.000000000 | 0.000339131 |
| _146_ | Scrophularoside A5 | 76.4 | 6.06562 | C_33_H_42_O_17_ | 691.2254891 | 0.000000000 | 0.000173776 |
| _147_ | Traumatic Acid | 76.4 | 8.09913 | C_12_H_20_O_4_ | 227.1285928 | 0.001007403 | 0.000054808 |
| _148_ | 3,5,25-Trihydroxyergostan-6-one | 76.3 | 10.53595 | C_19_H_30_O_3_ | 339.2528166 | 0.000839039 | 0.000039410 |
| _149_ | Chasmanthin | 76.3 | 6.42812 | C_20_H_22_O_7_ | 355.1188194 | 0.000000000 | 0.000073413 |
| _150_ | Palatiferin A | 76.3 | 6.96165 | C_21_H_20_O_8_ | 433.1494323 | 0.000000000 | 0.000122489 |
| _151_ | 1,4-Cyclohexanedicarboxylic acid | 76.2 | 5.71430 | C_8_H_12_O_4_ | 171.065585 | 0.007698438 | 0.000316690 |
| _152_ | Carpinontriol B | 76.2 | 6.32085 | C_19_H_20_O_6_ | 345.133182 | 0.000000000 | 0.000264606 |
| _153_ | Ala Val | 75.9 | 1.46048 | C_8_H_16_N_2_O_3_ | 187.1082399 | 0.000000000 | 0.000466249 |
| _154_ | Curculigoside | 75.8 | 6.07827 | C_22_H_26_O_11_ | 511.1459356 | 0.000000000 | 0.000413591 |
| _155_ | Qianhucoumarin A | 75.8 | 7.52853 | C_19_H_20_O_6_ | 325.1082019 | 0.000000000 | 0.000430893 |
| _156_ | Seguinoside D | 75.8 | 4.48817 | C_24_H_28_O_13_ | 505.1333202 | 0.000000000 | 0.000807206 |
| _157_ | Teucrin A | 75.8 | 6.33575 | C_19_H_20_O_6_ | 325.1082116 | 0.000000000 | 0.000460681 |
| _158_ | (+)-Puerol B 2''-O-glucoside | 75.7 | 5.46855 | C_24_H_26_O_10_ | 519.1512253 | 0.000000000 | 0.000385027 |
| _159_ | Sinapaldehyde | 75.6 | 6.92817 | C_11_H_12_O_4_ | 207.0658433 | 0.000131153 | 0.000340070 |
| _160_ | 4-Guanidinobutanoic acid | 75.5 | 0.87740 | C_5_H_11_N_3_O_2_ | 146.0924567 | 0.000113389 | 0.000563432 |
| _161_ | Asp Ile Leu | 75.5 | 5.56772 | C_16_H_29_N_3_O_6_ | 360.2126004 | 0.000000000 | 0.000182471 |
| _162_ | Australine | 75.5 | 4.48817 | C_8_H_15_NO_4_ | 226.0480294 | 0.000013610 | 0.000426977 |
| _163_ | Androstenediol | 75.4 | 12.49038 | C_19_H_30_O_2_ | 323.2580622 | 0.001189003 | 0.000000904 |
| _164_ | Durantoside II | 75.4 | 6.20513 | C_27_H_34_O_14_ | 563.1775229 | 0.000000000 | 0.000191120 |
| _165_ | Tinospinoside C | 75.2 | 5.00302 | C_27_H_36_O_12_ | 597.2193132 | 0.000000000 | 0.000785235 |
| _166_ | Methyl 5-O-feruloylquinate | 75.1 | 4.77442 | C_18_H_22_O_9_ | 427.1248679 | 0.000000000 | 0.000455829 |
| _167_ | Dehydroborapetoside B | 75 | 5.71430 | C_27_H_34_O_12_ | 595.2040343 | 0.000000000 | 0.010622201 |
| _168_ | Massarilactone B | 74.9 | 5.56772 | C_11_H_14_O_5_ | 227.0913367 | 0.000037060 | 0.004027569 |
| _169_ | Nakijiquinone H | 74.9 | 11.42547 | C_26_H_40_N_4_O_3_ | 474.3425541 | 0.000710269 | 0.000035661 |
| _170_ | Shikimic acid | 74.9 | 5.09273 | C_7_H_10_O_5_ | 173.0448987 | 0.000188187 | 0.000101012 |
| _171_ | Yomogiartemin | 74.9 | 6.02557 | C_17_H_20_O_7_ | 317.1031345 | 0.000000000 | 0.000811523 |
| _172_ | 6-Hydroxybenzofuran-2(3H)-one | 74.8 | 5.38867 | C_8_H_6_O_3_ | 359.0774337 | 0.000000000 | 0.000110648 |
| _173_ | Azadirachtin B | 74.8 | 7.14872 | C_33_H_42_O_14_ | 643.2404789 | 0.000000000 | 0.000183516 |
| _174_ | Chrysin 6-C-glucoside 8-C-arabinoside | 74.8 | 5.80587 | C_26_H_28_O_13_ | 549.1605195 | 0.000000000 | 0.001362523 |
| _175_ | Alpinin A | 74.6 | 6.23387 | C_20_H_24_O_7_ | 399.141622 | 0.000000000 | 0.000162525 |
| _176_ | Astraganoside | 74.6 | 4.83597 | C_23_H_28_O_11_ | 525.1615407 | 0.000000000 | 0.000602682 |
| _177_ | 10-Hydroxyoleoside 11-methyl ester | 74.4 | 3.99833 | C_17_H_24_O_12_ | 441.1017589 | 0.000000000 | 0.001136056 |
| _178_ | 5'-Methyl-Thioadenosine-sulfone | 74.4 | 1.39080 | C_11_H_15_N_5_O_4_S | 314.0917511 | 0.000000000 | 0.000925794 |
| _179_ | p-Hydroxybenzaldehyde | 74.3 | 6.01320 | C_7_H_6_O_2_ | 155.0702972 | 0.000147563 | 0.000209687 |
| _180_ | Mudanpioside J | 74.2 | 5.84538 | C_31_H_34_O_14_ | 675.1945058 | 0.000000000 | 0.000256222 |
| _181_ | Protocatechualdehyde | 74.1 | 4.07302 | C_7_H_6_O_3_ | 171.0652553 | 0.000120238 | 0.000733723 |
| _182_ | Ailanthoidol | 74 | 7.43555 | C_19_H_18_O_5_ | 307.0976608 | 0.000000000 | 0.000146529 |
| _183_ | Cuniloside | 74 | 9.02128 | C_26_H_40_O_10_ | 495.2585142 | 0.000000000 | 0.000143265 |
| _184_ | Riboflavin lumichrome | 74 | 6.42930 | C_12_H_10_N_4_O_2_ | 243.0875541 | 0.000000000 | 0.000091420 |
| _185_ | Uhdoside A | 74 | 6.45502 | C_32_H_36_O_13_ | 673.2150885 | 0.000000000 | 0.000193541 |
| _186_ | Dihydrodeoxy-8-epiaustdiol | 73.9 | 5.45582 | C_12_H_14_O_4_ | 267.0875078 | 0.000014603 | 0.000161686 |
| _187_ | Gentiobiose | 73.9 | 0.81412 | C_12_H_22_O_11_ | 325.1129432 | 0.023552337 | 0.000833251 |
| _188_ | Ile Glu Leu | 73.8 | 5.25280 | C_17_H_31_N_3_O_6_ | 374.2284609 | 0.000019727 | 0.000187340 |
| _189_ | L-Pyroglutamic acid | 73.8 | 1.01710 | C_5_H_7_NO_3_ | 130.0500484 | 0.004520743 | 0.026727906 |
| _190_ | 3-Methoxyflavone | 73.7 | 5.76715 | C_16_H_12_O_3_ | 297.0767829 | 0.000000000 | 0.000077404 |
| _191_ | 4-Hydroxybenzaldehyde rhamnoside | 73.7 | 4.95020 | C_13_H_16_O_6_ | 313.0930615 | 0.000067932 | 0.000567642 |
| _192_ | Ser Val Leu | 73.6 | 4.67038 | C_14_H_27_N_3_O_5_ | 318.2023503 | 0.000000000 | 0.000181488 |
| _193_ | 4-Oxododecanedioic acid | 73.5 | 6.84825 | C_12_H_20_O_5_ | 243.1236345 | 0.000334668 | 0.000045146 |
| _194_ | Paeonol | 73.5 | 7.29258 | C_9_H_10_O_3_ | 211.060794 | 0.000124358 | 0.000214570 |
| _195_ | Gibepyrone D | 73.4 | 5.22787 | C_10_H_10_O_4_ | 227.0912986 | 0.000299813 | 0.004183194 |
| _196_ | Kaempferol 3,7,4'-trimethylether | 73.4 | 7.04623 | C_18_H_16_O_6_ | 373.0929122 | 0.000000000 | 0.000052825 |
| _197_ | (-)-Medicocarpin | 73.3 | 5.76715 | C_22_H_24_O_9_ | 477.1406331 | 0.000000000 | 0.000219059 |
| _198_ | 2''-O-Rhamnosylicariside II | 73.2 | 6.59955 | C_33_H_40_O_14_ | 659.2357923 | 0.000000000 | 0.000728796 |
| _199_ | 5-O-Cinnamoylquinic acid | 73.2 | 5.76715 | C_16_H_18_O_7_ | 367.1036024 | 0.000000000 | 0.000357909 |
| _200_ | Calealactone C | 73.2 | 5.39823 | C_21_H_26_O_8_ | 371.148717 | 0.000000000 | 0.000107286 |
| _201_ | Diacetoxyscirpenol | 73.2 | 11.04563 | C_19_H_26_O_7_ | 347.1510775 | 0.084219250 | 0.000405799 |
| _202_ | indole-3-carboxylic acid beta-d-glucopyranosyl ester | 73.2 | 4.04965 | C_15_H_17_NO_7_ | 341.134285 | 0.000000000 | 0.000384541 |
| _203_ | Syringaldehyde | 73.1 | 6.01320 | C_9_H_10_O_4_ | 183.0653035 | 0.000232053 | 0.003121812 |
| _204_ | Picroside I | 73 | 7.12260 | C_24_H_28_O_11_ | 473.1456322 | 0.000000000 | 0.000576720 |
| _205_ | Uridine | 72.9 | 1.24040 | C_9_H_12_N_2_O_6_ | 279.0388842 | 0.000000000 | 0.000671597 |
| _206_ | 4-Quinolinecarboxylic acid | 72.8 | 1.44768 | C_10_H_7_NO_2_ | 172.0397195 | 0.000000000 | 0.000084234 |
| _207_ | 8-O-Acetyltorilolone | 72.8 | 11.95347 | C_17_H_26_O_4_ | 277.1798476 | 0.000892079 | 0.000065176 |
| _208_ | methyl 4-hydroxy-3,5-dimethoxybenzoate | 72.7 | 6.99715 | C_10_H_12_O_5_ | 213.07576 | 0.000016090 | 0.000129347 |
| _209_ | Succinic acid | 72.7 | 1.18057 | C_4_H_6_O_4_ | 117.01823 | 0.008288173 | 0.009978787 |
| _210_ | 5'-Deoxyadenosine | 72.6 | 1.80027 | C_10_H_13_N_5_O_3_ | 250.0944455 | 0.000000000 | 0.000284299 |
| _211_ | 6-Methoxymellein | 72.6 | 2.53740 | C_11_H_12_O_4_ | 253.0716553 | 0.000000000 | 0.000492814 |
| _212_ | apigenin | 72.6 | 6.18108 | C_15_H_10_O_5_ | 315.0489673 | 0.000000000 | 0.000031902 |
| _213_ | ferulic acid | 72.6 | 6.48967 | C_10_H_10_O_4_ | 177.0546425 | 0.000540053 | 0.000206317 |
| _214_ | gamma-Glutamylleucine | 72.6 | 4.22225 | C_11_H_20_N_2_O_5_ | 259.1299393 | 0.000000000 | 0.000052671 |
| _215_ | Gastrodin | 72.6 | 5.74718 | C_13_H_18_O_7_ | 251.0914011 | 0.000000000 | 0.000141202 |
| _216_ | Licoagroside B | 72.6 | 5.29798 | C_18_H_24_O_12_ | 453.1018819 | 0.000000000 | 0.000462729 |
| _217_ | Picropodophyllol | 72.6 | 5.41508 | C_22_H_26_O_8_ | 463.1616116 | 0.000000000 | 0.000200871 |
| _218_ | Deoxycytidine | 72.5 | 0.89640 | C_9_H_13_N_3_O_4_ | 228.0978699 | 0.000000000 | 0.001035016 |
| _219_ | Columbin | 72.4 | 6.84825 | C_20_H_22_O_6_ | 339.1238033 | 0.000000000 | 0.000356365 |
| _220_ | Menisdaurin | 72.4 | 5.63943 | C_14_H_19_NO_7_ | 296.1128611 | 0.000000000 | 0.000065998 |
| _221_ | Isoguanosine | 72.3 | 1.35912 | C_10_H_13_N_5_O_5_ | 284.0988866 | 0.000000000 | 0.001316245 |
| _222_ | 5,7,4'-Trimethoxyafzelechin | 71.9 | 6.74415 | C_18_H_20_O_5_ | 297.1132407 | 0.000000000 | 0.000079525 |
| _223_ | Excavatin M | 71.9 | 6.38688 | C_19_H_20_O_7_ | 359.1138445 | 0.000091057 | 0.000311658 |
| _224_ | Citrate | 71.8 | 3.70070 | C_6_H_8_O_7_ | 173.008447 | 0.000024413 | 0.001435755 |
| _225_ | Panowamycin B | 71.8 | 8.59642 | C_17_H_26_O_3_ | 311.2214478 | 0.000172725 | 0.000064476 |
| _226_ | Val Asn Ile | 71.7 | 4.59510 | C_15_H_28_N_4_O_5_ | 345.2131526 | 0.000000000 | 0.000097782 |
| _227_ | Cornoside | 71.6 | 6.01320 | C_14_H_20_O_8_ | 281.1018541 | 0.000000000 | 0.000154581 |
| _228_ | Triethyl phosphate | 71.6 | 6.81547 | C_6_H_15_O_4_P | 183.0783891 | 0.043617829 | 0.002433108 |
| _229_ | Asarylaldehyde | 71.4 | 7.37253 | C_10_H_12_O_4_ | 179.0703007 | 0.000000000 | 0.000049750 |
| _230_ | Senkyunolide G | 71.4 | 7.38318 | C_12_H_16_O_3_ | 253.1075399 | 0.001205173 | 0.000308103 |
| _231_ | Ceanothic acid acetate | 71.3 | 7.93097 | C_32_H_48_O_6_ | 529.3500627 | 0.000000000 | 0.000603562 |
| _232_ | putrescine | 71.3 | 0.60537 | C_4_H_12_N_2_ | 89.1079824 | 0.000000000 | 0.000169278 |
| _233_ | Cucurbitacin IIA | 71.2 | 7.02072 | C_32_H_50_O_8_ | 545.3450898 | 0.000000000 | 0.000417830 |
| _234_ | Thunberginol H | 71.2 | 7.01998 | C_17_H_16_O_5_ | 281.0815552 | 0.000204925 | 0.000074741 |
| _235_ | ()-Lariciresinol | 71.1 | 6.34842 | C_20_H_24_O_6_ | 405.1556267 | 0.000000000 | 0.000118746 |
| _236_ | 1,11-Undecanedicarboxylic acid | 71 | 9.00345 | C_13_H_24_O_4_ | 243.1599963 | 0.001220207 | 0.000066861 |
| _237_ | Ile Ile Asp | 71 | 4.36872 | C_16_H_29_N_3_O_6_ | 360.2127489 | 0.000000000 | 0.000085969 |
| _238_ | Sanshodiol | 71 | 6.53442 | C_20_H_22_O_6_ | 403.1399678 | 0.000000000 | 0.000420385 |
| _239_ | Tecomelloside | 71 | 5.27270 | C_24_H_30_O_13_ | 525.1614557 | 0.000000000 | 0.000800118 |
| _240_ | Ethyl alpha-D-fructofuranoside | 70.9 | 2.13838 | C_8_H_16_O_6_ | 250.1284652 | 0.000000000 | 0.000140381 |
| _241_ | Peujaponiside | 70.9 | 5.04040 | C_25_H_34_O_14_ | 539.1774094 | 0.000000000 | 0.000265054 |
| _242_ | Cordycepin | 70.6 | 1.80345 | C_10_H_13_N_5_O_3_ | 252.1090289 | 0.000000000 | 0.010586218 |
| _243_ | Dihydroresveratrol 3-O-glucoside | 70.6 | 6.40517 | C_20_H_24_O_8_ | 357.1330459 | 0.000000000 | 0.000338483 |
| _244_ | Retusin | 70.5 | 6.49458 | C_19_H_18_O_7_ | 393.0747269 | 0.000000000 | 0.000094080 |
| _245_ | 7,15-Dihydroxypodocarp-8(14)-en-13-one | 70.1 | 13.34197 | C_17_H_26_O_3_ | 277.1808698 | 0.000904205 | 0.000006455 |
| _246_ | Grayanotoxin XVIII | 70.1 | 8.59642 | C_20_H_32_O_4_ | 337.237138 | 0.000000000 | 0.000158491 |
| _247_ | swainsonine | 70 | 5.24620 | C_8_H_15_NO_3_ | 210.0529931 | 0.000019890 | 0.000209040 |
| _248_ | Fraxiresinol 1-O-glucoside | 69.9 | 4.79803 | C_27_H_34_O_13_ | 611.1987817 | 0.000000000 | 0.000726158 |
| _249_ | 2-(4-Hydroxy-3-methoxyphenyl)-7-methoxy-5-benzofuranpropanol | 69.8 | 6.53442 | C_19_H_20_O_5_ | 373.1293697 | 0.000000000 | 0.000289374 |
| _250_ | Cycloolivil | 69.8 | 5.74020 | C_20_H_24_O_7_ | 421.1507689 | 0.000000000 | 0.000405218 |
| _251_ | 4-Methylumbelliferyl acetate | 69.6 | 4.65707 | C_12_H_10_O_4_ | 251.0913865 | 0.000000000 | 0.000101388 |
| _252_ | alpha-Arbutin | 69.6 | 2.27365 | C_12_H_16_O_7_ | 255.0862668 | 0.000000000 | 0.000492387 |
| _253_ | Cnidioside B methyl ester | 69.6 | 5.60955 | C_19_H_24_O_10_ | 393.1193065 | 0.000000000 | 0.000239027 |
| _254_ | Pilosidine | 69.6 | 5.34983 | C_23_H_26_O_11_ | 523.1463025 | 0.000000000 | 0.000150623 |
| _255_ | Asterbatanoside A | 69.5 | 5.76715 | C_19_H_26_O_11_ | 411.1299509 | 0.000000000 | 0.000119374 |
| _256_ | Cnidioside A | 69.5 | 1.43515 | C_17_H_20_O_9_ | 413.1091106 | 0.000000000 | 0.001856199 |
| _257_ | Glu Ile Leu | 69.5 | 5.15377 | C_17_H_31_N_3_O_6_ | 374.2283372 | 0.000000000 | 0.000178734 |
| _258_ | Oxyresveratrol 3'-O-beta-D-glucopyranoside | 69.5 | 6.46408 | C_20_H_22_O_9_ | 371.1123811 | 0.000005276 | 0.000598362 |
| _259_ | Vanillylmandelic acid | 69.5 | 6.18108 | C_9_H_10_O_5_ | 219.0271223 | 0.000007026 | 0.000269753 |
| _260_ | Lucidone | 69.4 | 5.89728 | C_15_H_12_O_4_ | 301.0718113 | 0.000000000 | 0.000091549 |
| _261_ | Asperuloside | 69.3 | 4.06305 | C_18_H_22_O_11_ | 413.1083179 | 0.000000000 | 0.000195809 |
| _262_ | Grandidentoside | 69.3 | 6.86132 | C_21_H_28_O_10_ | 421.1507515 | 0.000000000 | 0.000055625 |
| _263_ | 2,4-DPD | 69.2 | 6.72057 | C_11_H_13_NO_4_ | 224.0917361 | 0.000004229 | 0.000128564 |
| _264_ | Axillarin 4'-glucuronide | 69.1 | 4.73443 | C_23_H_22_O_14_ | 503.0836426 | 0.000000000 | 0.001222675 |
| _265_ | alpha-guaiaconic acid | 69 | 6.09073 | C_20_H_20_O_5_ | 339.1236038 | 0.000000000 | 0.000048125 |
| _266_ | Scoparone | 69 | 5.90315 | C_11_H_10_O_4_ | 207.0652191 | 0.000000000 | 0.000279221 |
| _267_ | Bauhiniaside B | 68.9 | 6.62637 | C_22_H_26_O_9_ | 433.1507197 | 0.000000000 | 0.004694205 |
| _268_ | Heishuixiecaoline A | 68.9 | 11.76427 | C_17_H_24_O_3_ | 275.1653441 | 0.000397402 | 0.000004717 |
| _269_ | Ethyl-p-coumarate | 68.8 | 8.43790 | C_11_H_12_O_3_ | 193.0860487 | 0.000016804 | 0.000277435 |
| _270_ | 5,7-Dihydroxy-2-isopropylchromone | 68.7 | 5.81838 | C_12_H_12_O_4_ | 265.0718149 | 0.000000000 | 0.000555872 |
| _271_ | caffeic acid | 68.7 | 7.59155 | C_9_H_8_O_4_ | 181.0494673 | 0.000028159 | 0.000249829 |
| _272_ | Diosbulbin J | 68.7 | 6.38097 | C_19_H_22_O_8_ | 343.1175002 | 0.000000000 | 0.000153589 |
| _273_ | Geshoidin | 68.7 | 5.36090 | C_18_H_18_O_9_ | 343.0812261 | 0.000000000 | 0.000090239 |
| _274_ | N-Acetyl-phenylalanine | 68.6 | 5.77958 | C_11_H_13_NO_3_ | 206.0817929 | 0.000342180 | 0.000055415 |
| _275_ | Thr Tyr | 68.6 | 1.09453 | C_13_H_18_N_2_O_5_ | 283.12873 | 0.000000000 | 0.000135065 |
| _276_ | Ala Ser Phe | 68.5 | 3.95687 | C_15_H_21_N_3_O_5_ | 324.1553319 | 0.000000000 | 0.000147967 |
| _277_ | Methyloleoside | 68.3 | 4.28858 | C_17_H_24_O_11_ | 403.124652 | 0.000165467 | 0.002502578 |
| _278_ | N-Acetyl-leucine | 68.3 | 5.46855 | C_8_H_15_NO_3_ | 172.0972293 | 0.000075001 | 0.000047089 |
| _279_ | 9(S)-HODE | 68.2 | 11.55668 | C_18_H_32_O_3_ | 295.2279163 | 0.000407454 | 0.000010050 |
| _280_ | Aesculetin | 68.2 | 5.92712 | C_9_H_6_O_4_ | 211.060103 | 0.000000000 | 0.000057700 |
| _281_ | 5,7,4'-Tri-O-methylcatechin | 68 | 6.76995 | C_18_H_20_O_6_ | 313.1082736 | 0.000000000 | 0.000098723 |
| _282_ | Bergenin | 68 | 2.26120 | C_14_H_16_O_9_ | 373.0777571 | 0.000000000 | 0.000528578 |
| _283_ | Paeoniflorigenone | 67.9 | 5.37595 | C_17_H_18_O_6_ | 363.1088622 | 0.000000000 | 0.000206771 |
| _284_ | Xanthohumol B | 67.8 | 6.75712 | C_21_H_22_O_6_ | 369.1344303 | 0.000000000 | 0.000122733 |
| _285_ | 8,9-Didehydro-7-hydroxydolichodial | 67.6 | 5.89728 | C_10_H_12_O_3_ | 225.0765566 | 0.000300571 | 0.000206450 |
| _286_ | Asp Val Leu | 67.6 | 4.92690 | C_15_H_27_N_3_O_6_ | 346.1972418 | 0.000000000 | 0.000351426 |
| _287_ | Purine | 67.5 | 14.76140 | C_5_H_4_N_4_ | 138.0775186 | 0.007414948 | 0.000493044 |
| _288_ | Syringopicroside | 67.5 | 6.71853 | C_24_H_30_O_11_ | 515.1539517 | 0.000000000 | 0.000209582 |
| _289_ | 12-Hydroxy-2,3-dihydroeuparin | 67.4 | 6.75712 | C_13_H_14_O_4_ | 279.0873663 | 0.000203402 | 0.000154486 |
| _290_ | N-Lactoyl-phenylalanine | 67.4 | 5.82943 | C_12_H_15_NO_4_ | 238.1074313 | 0.000000000 | 0.000037785 |
| _291_ | 1-Nonanol | 67.3 | 11.11883 | C_9_H_20_O | 330.3366258 | 0.004399148 | 0.000012428 |
| _292_ | D-Aspartic acid | 67.3 | 0.77347 | C_4_H_7_NO_4_ | 134.0449286 | 0.004606687 | 0.000006793 |
| _293_ | Kakuol | 67.3 | 1.48558 | C_10_H_10_O_4_ | 239.0558446 | 0.000236226 | 0.000207155 |
| _294_ | Ala Ile Asp | 67.1 | 1.80345 | C_13_H_23_N_3_O_6_ | 318.1659745 | 0.000000000 | 0.000220151 |
| _295_ | Pantoyllactone glucoside | 67.1 | 4.26215 | C_12_H_20_O_8_ | 337.1140742 | 0.000000000 | 0.000249705 |
| _296_ | 3-Oxopentanedioic acid | 67 | 0.98200 | C_5_H_6_O_5_ | 191.0190043 | 0.002575676 | 0.342183400 |
| _297_ | Pendulone | 67 | 5.66200 | C_17_H_16_O_6_ | 361.0931226 | 0.000000000 | 0.000106650 |
| _298_ | Harmalan | 66.9 | 5.02835 | C_12_H_12_N_2_ | 229.0980104 | 0.000009707 | 0.000510617 |
| _299_ | 6alpha-Hydroxyandrost-4-ene-3,17-dione | 66.8 | 5.17682 | C_19_H_26_O_3_ | 347.160008 | 0.000000000 | 0.000164034 |
| _300_ | Albaspidin AP | 66.8 | 5.23390 | C_22_H_26_O_8_ | 417.1557085 | 0.000000000 | 0.000348598 |
| _301_ | Cassiaglycoside II | 66.8 | 5.66200 | C_25_H_32_O_14_ | 601.1776732 | 0.000000000 | 0.000287001 |
| _302_ | Di-O-methylbergenin | 66.8 | 1.33570 | C_16_H_20_O_9_ | 401.1090179 | 0.000000000 | 0.000648503 |
| _303_ | Rhinocerotinoic acid | 66.8 | 9.96640 | C_20_H_30_O_3_ | 355.168126 | 0.000922883 | 0.000027366 |
| _304_ | Tectoruside | 66.7 | 4.77442 | C_21_H_30_O_13_ | 471.1511202 | 0.000000000 | 0.000399004 |
| _305_ | 4-Methylcoumarin | 66.6 | 4.67038 | C_10_H_8_O_2_ | 161.0597376 | 0.000053596 | 0.000035236 |
| _306_ | METAMECONINE | 66.5 | 7.20093 | C_10_H_10_O_4_ | 193.0501012 | 0.000359341 | 0.000237455 |
| _307_ | Ala Val Val | 66.4 | 3.69575 | C_13_H_25_N_3_O_4_ | 288.1915376 | 0.000044843 | 0.000126548 |
| _308_ | Chrysosplenetin | 66.4 | 5.34983 | C_19_H_18_O_8_ | 419.0986794 | 0.000000000 | 0.000315684 |
| _309_ | Zeylenone | 66.4 | 5.63943 | C_21_H_18_O_7_ | 415.1384495 | 0.000000000 | 0.000287270 |
| _310_ | Ala Leu Glu | 66.3 | 1.42503 | C_14_H_25_N_3_O_6_ | 332.1815915 | 0.000000000 | 0.000144358 |
| _311_ | 15-Deoxypulic acid | 66.2 | 12.39893 | C_20_H_26_O_4_ | 375.1822802 | 0.015611162 | 0.000005576 |
| _312_ | Icariside B5 | 66.2 | 5.09273 | C_19_H_32_O_8_ | 387.202696 | 0.000000000 | 0.000690530 |
| _313_ | Glochicoccin D | 66.1 | 5.79292 | C_21_H_24_O_10_ | 435.129861 | 0.000000000 | 0.000339574 |
| _314_ | Piceoside | 66.1 | 5.97720 | C_14_H_18_O_7_ | 597.2180635 | 0.000000000 | 0.000897749 |
| _315_ | bufalin | 66 | 11.25928 | C_24_H_34_O_4_ | 387.256258 | 0.002131848 | 0.000162193 |
| _316_ | Isovanillin | 66 | 5.87848 | C_8_H_8_O_3_ | 153.054671 | 0.001248875 | 0.002159506 |
| _317_ | 2,7-Dimethyl-1,4-dihydroxynaphthalene  1-O-glucoside | 65.9 | 5.90315 | C_18_H_22_O_7_ | 333.1331925 | 0.000000000 | 0.000088057 |
| _318_ | Hamaudol | 65.8 | 9.77432 | C_15_H_16_O_5_ | 259.0964766 | 0.000000000 | 0.000028771 |
| _319_ | Sarmentosin | 65.8 | 5.16423 | C_11_H_17_NO_7_ | 240.0866422 | 0.000000000 | 0.000074536 |
| _320_ | Senkyunolide H | 65.8 | 7.38318 | C_12_H_16_O_4_ | 223.097232 | 0.000065082 | 0.000432982 |
| _321_ | 7-Hydroxyflavanone-glucoside | 65.7 | 5.61530 | C_21_H_22_O_8_ | 435.1650204 | 0.000000000 | 0.002317243 |
| _322_ | Curcumenol | 65.7 | 11.63553 | C_15_H_22_O_2_ | 233.154474 | 0.019563288 | 0.000544793 |
| _323_ | Eupalinolide H | 65.7 | 6.27177 | C_22_H_28_O_8_ | 385.1644265 | 0.000000000 | 0.000172701 |
| _324_ | Alanylisoleucine | 65.6 | 2.04145 | C_9_H_18_N_2_O_3_ | 201.1239686 | 0.000000000 | 0.000244182 |
| _325_ | Licraside | 65.6 | 5.59715 | C_26_H_30_O_13_ | 549.1621372 | 0.000000000 | 0.000501836 |
| _326_ | 3-Ethoxy-4-hydroxybenzaldehyde | 65.5 | 5.00302 | C_9_H_10_O_3_ | 211.0608121 | 0.000474961 | 0.001360304 |
| _327_ | Carabrolactone A | 65.5 | 12.63418 | C_15_H_22_O_5_ | 315.1802446 | 0.008646793 | 0.000330479 |
| _328_ | Ginnalin B | 65.4 | 1.89060 | C_13_H_16_O_9_ | 315.072352 | 0.000000000 | 0.002370245 |
| _329_ | Deacetylgedunin | 65.3 | 12.93817 | C_26_H_32_O_6_ | 421.203301 | 0.003112350 | 0.000111714 |
| _330_ | Hexyl glucoside | 65.3 | 7.38318 | C_12_H_24_O_6_ | 245.1393267 | 0.000000000 | 0.000135068 |
| _331_ | androstane-3,6,17-triol | 65.2 | 10.43478 | C_19_H_32_O_3_ | 353.2334576 | 0.000000000 | 0.000048430 |
| _332_ | 7-methoxy-6-(1,2,3-trihydroxy-3-methylbutyl)chromen-2-one | 65.1 | 7.04637 | C_15_H_18_O_6_ | 295.1152558 | 0.000000000 | 0.000173889 |
| _333_ | arecoline | 65.1 | 6.82283 | C_8_H_13_NO_2_ | 200.0923776 | 0.000018844 | 0.000055641 |
| _334_ | Herbarin | 65.1 | 7.08280 | C_16_H_16_O_6_ | 285.0768638 | 0.000000000 | 0.000000917 |
| _335_ | Icosanedioic acid | 65.1 | 12.38410 | C_20_H_38_O_4_ | 365.2662615 | 0.000844438 | 0.000000000 |
| _336_ | Leu Val Ile | 65.1 | 5.40992 | C_17_H_33_N_3_O_4_ | 344.2542516 | 0.000000000 | 0.000310950 |
| _337_ | (-)-Syringaresnol-4-O  -beta-D-apiofuranos | 65 | 5.75342 | C_33_H_44_O_17_ | 693.2410383 | 0.000000000 | 0.000435047 |
| _338_ | 8-Hydroxypinoresinol-acetat | 65 | 6.09073 | C_28_H_34_O_13_ | 577.1932826 | 0.000000000 | 0.000259139 |
| _339_ | Creoside III | 65 | 4.44868 | C_18_H_24_O_9_ | 429.1404094 | 0.000000000 | 0.000111060 |
| _340_ | Salvianolic acid D | 64.9 | 5.38442 | C_20_H_18_O_10_ | 451.1235419 | 0.000000000 | 0.000240514 |
| _341_ | 5-O-Methylvisammioside | 64.7 | 5.36332 | C_22_H_28_O_10_ | 433.1505381 | 0.000000000 | 0.000091482 |
| _342_ | Pantothenic acid | 64.7 | 2.84053 | C_9_H_17_NO_5_ | 218.10303 | 0.000006864 | 0.001815859 |
| _343_ | Rishirilide B | 64.7 | 6.03872 | C_21_H_24_O_6_ | 417.1557929 | 0.000000000 | 0.000134409 |
| _344_ | 2-(4-Hydroxyphenyl)-6-methy  l-2,3-dihydro-4H-pyran-4-one | 64.4 | 6.10400 | C_12_H_12_O_3_ | 249.0767627 | 0.000157906 | 0.000105761 |
| _345_ | Harman | 64.4 | 4.90142 | C_12_H_10_N_2_ | 183.0915999 | 0.000020114 | 0.000108285 |
| _346_ | Delta-Gluconolactone | 64.3 | 0.85005 | C_6_H_10_O_6_ | 223.0454903 | 0.000010018 | 0.000361850 |
| _347_ | 5-Methoxyauaranetin | 64.1 | 6.82283 | C_21_H_22_O_8_ | 447.1299886 | 0.000000000 | 0.000337520 |
| _348_ | Picrotoxinin | 64.1 | 6.90127 | C_15_H_16_O_6_ | 273.0769558 | 0.000000000 | 0.000081461 |
| _349_ | Diderroside | 64 | 2.20935 | C_19_H_28_O_13_ | 463.1461246 | 0.000000000 | 0.000598833 |
| _350_ | p-Coumaric Acid Ethyl Ester | 64 | 8.44160 | C_11_H_12_O_3_ | 191.0707596 | 0.000766341 | 0.001584094 |
| _351_ | Dianthoside | 63.9 | 6.00025 | C_12_H_16_O_8_ | 269.0668147 | 0.000000000 | 0.000098407 |
| _352_ | Didymin | 63.9 | 6.02557 | C_28_H_34_O_14_ | 593.1885676 | 0.000000000 | 0.000301505 |
| _353_ | Longicaulenone | 63.9 | 12.64742 | C_12_H_18_O_4_ | 227.1277714 | 0.035741768 | 0.001112716 |
| _354_ | Cnicin | 63.8 | 11.04563 | C_20_H_26_O_7_ | 377.1614463 | 0.001703795 | 0.000016007 |
| _355_ | (-)-Lyoniresinol 9'-O-glucoside | 63.7 | 5.36332 | C_28_H_38_O_13_ | 627.230458 | 0.000000000 | 0.000416103 |
| _356_ | 2-Phenylethyl b-D-glucopyranoside | 63.7 | 4.24980 | C_14_H_20_O_6_ | 249.1121294 | 0.000000000 | 0.000074459 |
| _357_ | Borapetoside F | 63.7 | 6.03872 | C_27_H_34_O_11_ | 579.209017 | 0.000000000 | 0.000893153 |
| _358_ | Calophymembranside B | 63.7 | 4.93758 | C_20_H_30_O_12_ | 461.1669764 | 0.000000000 | 0.000559221 |
| _359_ | Isogibberellin A3 | 63.7 | 5.65157 | C_19_H_22_O_6_ | 311.1277204 | 0.000000000 | 0.000070675 |
| _360_ | Methyl gallate | 63.7 | 4.39333 | C_8_H_8_O_5_ | 183.0293562 | 0.000097700 | 0.000058122 |
| _361_ | Pinobanksin 3-O-propanoate | 63.7 | 5.53352 | C_18_H_16_O_6_ | 373.0929375 | 0.000000000 | 0.000420899 |
| _362_ | Senkyunolide N | 63.6 | 7.57893 | C_12_H_18_O_4_ | 225.1129202 | 0.000498718 | 0.000090601 |
| _363_ | undecylenic acid | 63.6 | 9.37928 | C_11_H_20_O_2_ | 229.1442561 | 0.000437061 | 0.000101236 |
| _364_ | 9-Oxo-10,11-dehydroageraphorone | 63.5 | 4.14518 | C_15_H_20_O_2_ | 277.1182157 | 0.000000000 | 0.000257064 |
| _365_ | Neposide | 63.3 | 5.82943 | C_19_H_22_O_8_ | 361.1279606 | 0.000000000 | 0.000295273 |
| _366_ | 3'-Deoxy-4-O-methylepisappanol | 63.2 | 5.92250 | C_17_H_18_O_5_ | 347.1137972 | 0.000000000 | 0.000225612 |
| _367_ | Olivil 4'-O-Glucoside | 63 | 5.65157 | C_26_H_34_O_12_ | 503.191217 | 0.000000000 | 0.000183045 |
| _368_ | (1R,3R,4S,5R)-1,3,4-trihydroxy  -5-[(E)-3-(4-hydroxyphenyl)prop-2-enoyl]oxycyclohexane-1-carboxylic acid | 62.9 | 4.56432 | C_16_H_18_O_8_ | 337.0931711 | 0.000000000 | 0.002108541 |
| _369_ | Arabinofuranosyluracil | 62.9 | 1.03813 | C_9_H_12_N_2_O_6_ | 243.0622634 | 0.000476550 | 0.000372090 |
| _370_ | Avocadyne Acetate | 62.9 | 13.34872 | C_19_H_34_O_4_ | 368.2770892 | 0.000269209 | 0.000000000 |
| _371_ | Leu Leu Gly | 62.9 | 4.04965 | C_14_H_27_N_3_O_4_ | 302.2074263 | 0.000000000 | 0.000000000 |
| _372_ | Ribothymidine | 62.9 | 1.61190 | C_10_H_14_N_2_O_6_ | 257.0777328 | 0.000010572 | 0.000077513 |
| _373_ | Anhydrobrazilic acid | 62.8 | 3.28803 | C_12_H_10_O_5_ | 235.0600784 | 0.000012111 | 0.003374385 |
| _374_ | Cyclo(Leu-Leu) | 62.8 | 5.49533 | C_12_H_22_N_2_O_2_ | 511.3503641 | 0.146843385 | 0.002537756 |
| _375_ | Rhodojaponin II | 62.8 | 9.96640 | C_22_H_34_O_7_ | 445.1999962 | 0.008332351 | 0.000218574 |
| _376_ | 5-Hydroxy-2-methylchromone | 62.6 | 6.44060 | C_10_H_8_O_3_ | 209.0808594 | 0.000005834 | 0.000235019 |
| _377_ | Puerol A | 62.6 | 7.16707 | C_17_H_14_O_5_ | 331.1176368 | 0.000000000 | 0.000130071 |
| _378_ | Alismoxide | 62.4 | 5.21557 | C_15_H_26_O_2_ | 302.2073718 | 0.000000000 | 0.000197703 |
| _379_ | Luteolin-7-O-alpha-L-  arabinopyranosyl (1->6)-  beta-D-glucopyranoside | 62.4 | 5.57060 | C_26_H_28_O_15_ | 561.1249357 | 0.000000000 | 0.000397236 |
| _380_ | N,N-Dimethyldecylamine oxide | 62.4 | 7.64047 | C_12_H_27_NO | 202.216537 | 0.007411895 | 0.000485825 |
| _381_ | 13-Oxo-9E,11E-octadecadienoic acid | 62.3 | 8.05120 | C_18_H_30_O_3_ | 277.2161252 | 0.000296617 | 0.000609554 |
| _382_ | Podoverin A | 62.3 | 5.04578 | C_21_H_20_O_7_ | 417.1540385 | 0.000000000 | 0.000245446 |
| _383_ | Quercetin 3,5,7,3,4-pentamethyl ether | 62.3 | 8.45477 | C_20_H_20_O_7_ | 803.2568355 | 0.000000000 | 0.000360880 |
| _384_ | 1-(4-hydroxyphenyl)-3-[(2R,3R,4S,5S,6R)-3,4,5-trihydroxy-6-(hydroxymethyl)oxan-2-yl]oxypropan-1-one | 62.2 | 4.51218 | C_15_H_20_O_8_ | 327.1086705 | 0.000011001 | 0.002741163 |
| _385_ | 3',4',7-Trimethoxyquercetin | 62.2 | 5.05347 | C_18_H_16_O_7_ | 389.0879763 | 0.000073111 | 0.000119200 |
| _386_ | 6'-O-Cinnamoylharpagide | 62.2 | 7.99535 | C_24_H_30_O_11_ | 475.1611654 | 0.000000000 | 0.000052914 |
| _387_ | Purpurogallin | 62.2 | 3.88072 | C_11_H_8_O_5_ | 265.0354593 | 0.000000000 | 0.000060561 |
| _388_ | prostaglandin F2alpha | 62.1 | 11.51542 | C_20_H_34_O_5_ | 377.2298097 | 0.001240589 | 0.000020856 |
| _389_ | 10-Hydroxyoleuropein | 62 | 5.01497 | C_25_H_32_O_14_ | 555.17245 | 0.000000000 | 0.000877166 |
| _390_ | Ile Asn | 62 | 0.99827 | C_10_H_19_N_3_O_4_ | 246.144871 | 0.000000000 | 0.001774302 |
| _391_ | (E)-6-O-(p-coumaroyl)scandoside methyl ester | 61.9 | 8.05120 | C_26_H_30_O_13_ | 515.1548694 | 0.000000000 | 0.000452941 |
| _392_ | Aigialomycin D | 61.9 | 6.74567 | C_18_H_22_O_6_ | 317.1382768 | 0.000000000 | 0.000055133 |
| _393_ | Picraquassioside B | 61.9 | 4.91063 | C_19_H_24_O_11_ | 473.1302638 | 0.000000000 | 0.000165369 |
| _394_ | D-mannitol | 61.7 | 0.78920 | C_6_H_14_O_6_ | 181.0710689 | 0.011720358 | 0.000065272 |
| _395_ | Nagilactone C | 61.7 | 7.42288 | C_19_H_22_O_7_ | 327.1226248 | 0.000000000 | 0.000162887 |
| _396_ | De-O-methyllasiodiplodin | 61.6 | 11.09848 | C_16_H_22_O_4_ | 277.1444898 | 0.012560294 | 0.000390510 |
| _397_ | Ile Ile Gly | 61.6 | 4.34497 | C_14_H_27_N_3_O_4_ | 302.2073933 | 0.000000000 | 0.000089015 |
| _398_ | Periplanetin | 61.6 | 5.44275 | C_13_H_16_O_7_ | 265.0717717 | 0.000000000 | 0.000145854 |
| _399_ | Caffeic acid 4-O-glucuronide | 61.5 | 5.22787 | C_15_H_16_O_10_ | 339.0710536 | 0.000000000 | 0.000218730 |
| _400_ | 1-Oxo-4-hydroxy-2-en-4-ethylcyclohexa-5,8-olide | 61.4 | 3.34265 | C_8_H_8_O_4_ | 167.0342252 | 0.001136393 | 0.000211703 |
| _401_ | N6-benzyl-7H-purine-2,6-diamine | 61.3 | 1.01710 | C_12_H_12_N_6_ | 258.1448601 | 0.000000000 | 0.000727842 |
| _402_ | 5-Hydroxymethyl-2-furaldehyde | 61.2 | 5.52003 | C_6_H_6_O_3_ | 311.0774125 | 0.000000000 | 0.001420428 |
| _403_ | Cryptostrobin | 61.2 | 6.58722 | C_16_H_14_O_4_ | 315.0876806 | 0.000000000 | 0.000037502 |
| _404_ | 5-Acetoxy-7-hydroxyflavone | 61.1 | 6.67842 | C_17_H_12_O_5_ | 341.0668275 | 0.000000000 | 0.000038059 |
| _405_ | 6,8-Dimethyl-4-hydroxycoumarin | 61.1 | 2.86667 | C_11_H_10_O_3_ | 189.0551651 | 0.000005810 | 0.000063082 |
| _406_ | Phellolactone | 61.1 | 4.33255 | C_13_H_14_O_8_ | 331.1022541 | 0.000000000 | 0.000301733 |
| _407_ | scopoletin | 61 | 4.48817 | C_10_H_8_O_4_ | 237.0403606 | 0.000000000 | 0.000195277 |
| _408_ | (1S,3R,4R,5R)-1,3,4-trihydroxy-5-[(E)-3-(4-hydroxyphenyl)prop-2-enoyl]oxycyclohexane-1-carboxylic acid | 60.9 | 4.55800 | C_16_H_18_O_8_ | 339.1074905 | 0.000000000 | 0.000212114 |
| _409_ | 2-Propylpiperidine | 60.9 | 14.85198 | C_8_H_17_N | 128.1435483 | 0.011388295 | 0.000757220 |
| _410_ | Apigenin 7-[rhamnosyl-(1->2)-galacturonide] | 60.9 | 5.50695 | C_27_H_28_O_15_ | 591.1360578 | 0.000000000 | 0.000369679 |
| _411_ | Curvulinic acid | 60.7 | 1.99550 | C_10_H_10_O_5_ | 211.0600918 | 0.000000000 | 0.000701523 |
| _412_ | Wushanicaritin | 60.7 | 5.12920 | C_21_H_22_O_7_ | 431.1348188 | 0.000000000 | 0.000199928 |
| _413_ | Cerberidol | 60.6 | 8.55743 | C_9_H_16_O_3_ | 343.2126127 | 0.000000000 | 0.000039133 |
| _414_ | Megastigm-7-ene-3,4,6,9-tetrol | 60.5 | 6.11135 | C_13_H_24_O_4_ | 209.1536149 | 0.000133278 | 0.000075198 |
| _415_ | Methyl 4-hydroxy-3-methoxycinnamate | 60.5 | 6.92525 | C_11_H_12_O_4_ | 209.0808694 | 0.000117137 | 0.000613792 |
| _416_ | Tuberosin | 60.4 | 8.28302 | C_20_H_18_O_5_ | 383.1135852 | 0.000000000 | 0.000146659 |
| _417_ | emodin | 60.3 | 6.54675 | C_15_H_10_O_5_ | 269.0456508 | 0.001230773 | 0.000021792 |
| _418_ | 1,7-Dihydroxyxanthone (Euxanthone, Purrenone) | 60.2 | 6.16908 | C_13_H_8_O_4_ | 273.0405706 | 0.000000000 | 0.000869375 |
| _419_ | 12-O-Methylsordariol | 60.2 | 12.64742 | C_13_H_18_O_4_ | 271.1539165 | 0.030742151 | 0.001037511 |
| _420_ | Cytosine | 60.2 | 0.84338 | C_4_H_5_N_3_O | 112.0509432 | 0.000126445 | 0.005363094 |
| _421_ | Neoagarobiose | 60.2 | 2.39402 | C_12_H_20_O_10_ | 345.0805165 | 0.000000000 | 0.000600515 |
| _422_ | Poliothyrsoside | 60.2 | 7.07130 | C_20_H_22_O_9_ | 405.119267 | 0.000000000 | 0.000476591 |
| _423_ | 2,4-Dihydroxy-6-methoxy-3-formylacetophenone | 60.1 | 3.97123 | C_10_H_10_O_5_ | 255.0509573 | 0.000000000 | 0.000374568 |
| _424_ | 6-O-Ethyltetradymodiol | 60 | 7.33080 | C_17_H_26_O_3_ | 323.1863826 | 0.000094431 | 0.000144078 |
| _425_ | Eucomic acid | 60 | 1.46952 | C_11_H_12_O_6_ | 241.0706292 | 0.000000000 | 0.000264321 |
| _426_ | 1,11b-Dihydro-11b-hydroxymaackiain | 59.9 | 7.68317 | C_16_H_14_O_6_ | 301.0718104 | 0.000000000 | 0.000080569 |
| _427_ | Koaburaside monomethyl ether | 59.8 | 4.84292 | C_15_H_22_O_9_ | 347.133574 | 0.000000000 | 0.000278198 |
| _428_ | Catechin 7-arabinofuranoside | 59.7 | 6.10400 | C_20_H_22_O_10_ | 421.1143578 | 0.000000000 | 0.000080904 |
| _429_ | 9,16-Dioxo-10,12,14-octadecatrienoic acid | 59.6 | 9.48347 | C_18_H_26_O_4_ | 305.1759023 | 0.000000000 | 0.000218807 |
| _430_ | Niazirin | 59.6 | 5.96468 | C_14_H_17_NO_5_ | 312.1440625 | 0.000000000 | 0.000201468 |
| _431_ | D-Glucosamine | 59.5 | 1.33570 | C_6_H_13_NO_5_ | 160.060749 | 0.000111908 | 0.000114247 |
| _432_ | 3-HYDROXYBENZOATE | 59.4 | 4.76057 | C_7_H_6_O_3_ | 173.0004281 | 0.000068398 | 0.000032891 |
| _433_ | Crobarbatine | 59.4 | 9.01718 | C_15_H_21_NO_5_ | 276.1240845 | 0.000000000 | 0.000102910 |
| _434_ | Helenalin A | 59.4 | 6.02557 | C_15_H_18_O_4_ | 307.1187786 | 0.000000000 | 0.000035526 |
| _435_ | 1-(4-Hydroxybenzoyl)glucose | 59.3 | 4.35388 | C_13_H_16_O_8_ | 299.0772071 | 0.000000000 | 0.000408157 |
| _436_ | 3-formyl-4-oxo-4H-chromen-7-yl acetate | 59.3 | 6.38688 | C_12_H_8_O_5_ | 231.0297011 | 0.000000000 | 0.000024546 |
| _437_ | Taiwapyrone | 59.1 | 7.29258 | C_10_H_14_O_4_ | 179.070677 | 0.000063873 | 0.000261415 |
| _438_ | Americanol A | 59 | 7.08280 | C_18_H_18_O_6_ | 375.1086442 | 0.000000000 | 0.000341281 |
| _439_ | Baicalin methyl ester | 59 | 5.81838 | C_22_H_20_O_11_ | 459.0937748 | 0.000000000 | 0.000091074 |
| _440_ | Lappaol A | 59 | 7.31788 | C_30_H_32_O_9_ | 581.2034757 | 0.000000000 | 0.000558256 |
| _441_ | Loganetin | 59 | 5.50932 | C_11_H_16_O_5_ | 211.0965077 | 0.000028828 | 0.000039314 |
| _442_ | 6-Hydroxy-7-methoxydihydroligustilide | 58.9 | 9.19667 | C_13_H_18_O_4_ | 219.1022298 | 0.000382226 | 0.000011249 |
| _443_ | 4-Methyl-6,7-dihydroxycoumarin | 58.8 | 4.49908 | C_10_H_8_O_4_ | 193.0496565 | 0.000015939 | 0.000112077 |
| _444_ | 6-Methoxy-4-methylcoumarin | 58.8 | 6.91255 | C_11_H_10_O_3_ | 191.0702993 | 0.000006761 | 0.000044756 |
| _445_ | picrotin | 58.8 | 6.41438 | C_15_H_18_O_7_ | 309.0981584 | 0.000000000 | 0.000027981 |
| _446_ | Plumbagin | 58.8 | 7.68317 | C_11_H_8_O_3_ | 187.0395006 | 0.000000000 | 0.000018428 |
| _447_ | Citropten | 58.7 | 4.67040 | C_11_H_10_O_4_ | 251.0560826 | 0.000000000 | 0.001799709 |
| _448_ | 4'-Hydroxy-5,7,3'-trimethoxyflavone | 58.6 | 6.29582 | C_18_H_16_O_6_ | 327.0875335 | 0.000000000 | 0.000059685 |
| _449_ | 7-Methoxycoumarin | 58.6 | 5.42267 | C_10_H_8_O_3_ | 209.0808583 | 0.000012852 | 0.000369723 |
| _450_ | ethyl 2-(2-acetyl-3,5-dihydroxyphenyl)acetate | 58.6 | 8.42530 | C_12_H_14_O_5_ | 239.0903446 | 0.000258665 | 0.000039326 |
| _451_ | erythro-Guaiacylglycerol-beta-O-4'-dehydrodisinapyl ether | 58.5 | 6.96707 | C_31_H_36_O_11_ | 583.2192198 | 0.000000000 | 0.000816163 |
| _452_ | 7-O-Methylepimedonin G | 58.3 | 6.08625 | C_21_H_20_O_6_ | 401.1594654 | 0.000000000 | 0.000426648 |
| _453_ | Euscaphin B | 58.2 | 9.95887 | C_14_H_26_O_4_ | 241.179803 | 0.001551230 | 0.000324139 |
| _454_ | Sutherlandin trans-p-coumarate | 58.1 | 6.85317 | C_20_H_23_NO_9_ | 386.1232712 | 0.000000000 | 0.000186765 |
| _455_ | cinnamaldehyde | 58 | 5.28830 | C_9_H_8_O | 133.0649241 | 0.000279043 | 0.000180713 |
| _456_ | 6-Methylcoumarin | 57.9 | 5.91505 | C_10_H_8_O_2_ | 161.0597353 | 0.000020163 | 0.000028678 |
| _457_ | Teucryeminone | 57.8 | 6.76995 | C_24_H_28_O_9_ | 505.1720133 | 0.000000000 | 0.000096561 |
| _458_ | (R,E)-Deca-2-ene-4,6-diyne-1,8-diol | 57.7 | 7.55440 | C_10_H_12_O_2_ | 209.0815616 | 0.000040033 | 0.000032817 |
| _459_ | 5,6,7,3',4',5'-Hexamethoxyflavanone | 57.7 | 6.73378 | C_21_H_24_O_8_ | 427.1365299 | 0.000000000 | 0.000439462 |
| _460_ | Glu Tyr | 57.7 | 1.16117 | C_14_H_18_N_2_O_6_ | 311.1236955 | 0.000000000 | 0.000276665 |
| _461_ | Glycycoumarin | 57.7 | 6.27177 | C_21_H_20_O_6_ | 401.1593852 | 0.000000000 | 0.000269309 |
| _462_ | Phthalic acid | 57.7 | 4.64395 | C_8_H_6_O_4_ | 165.0186244 | 0.001058984 | 0.000533007 |
| _463_ | Agrimonolide 6-O-glucoside | 57.6 | 4.69660 | C_24_H_28_O_10_ | 521.1668963 | 0.000000000 | 0.000422018 |
| _464_ | Pistaciamide | 57.5 | 4.08483 | C_11_H_11_NO_4_ | 239.1025183 | 0.000000000 | 0.000000000 |
| _465_ | Corchoionol C | 57.5 | 9.15522 | C_13_H_20_O_3_ | 269.1392641 | 0.002138697 | 0.000160732 |
| _466_ | 8-O-Acetylharpagide | 57.5 | 4.84780 | C_17_H_26_O_11_ | 405.1403619 | 0.000102232 | 0.002309991 |
| _467_ | 13-Hydroxy-9Z,11E-octadecadienoic acid | 57.4 | 13.30187 | C_18_H_32_O_3_ | 277.2171717 | 0.000402947 | 0.000003881 |
| _468_ | Madurensine | 57.3 | 4.89008 | C_18_H_25_NO_6_ | 396.1401664 | 0.000000000 | 0.000128715 |
| _469_ | N-[(1S,4S,5S)-2-[2-(4-methoxyphenyl)acetyl]-2-azabicyclo[2.2.1]heptan-5-yl]methanesulfonamide | 57.3 | 5.39823 | C_16_H_22_N_2_O_4_S | 339.1371109 | 0.000000000 | 0.000087011 |
| _470_ | farnesol | 57.3 | 4.86613 | C_15_H_26_O | 245.1859672 | 0.000000000 | 0.000553898 |
| _471_ | Ethyl-beta-glucuronide | 57 | 1.03813 | C_8_H_14_O_7_ | 221.0662375 | 0.007620706 | 0.000895712 |
| _472_ | 1-[4-(4-hydroxybenzoyl)piperazin-1-yl]ethanone | 57 | 4.87750 | C_13_H_16_N_2_O_3_ | 249.1232672 | 0.000000000 | 0.000168385 |
| _473_ | 5-Hydroxy-1-tetralone | 56.9 | 5.60955 | C_10_H_10_O_2_ | 207.065873 | 0.000034377 | 0.000049028 |
| _474_ | Uracil | 56.9 | 1.23697 | C_4_H_4_N_2_O_2_ | 113.0349085 | 0.000675162 | 0.001290075 |
| _475_ | Erythrinin G | 56.9 | 7.39787 | C_20_H_18_O_6_ | 387.1437898 | 0.000000000 | 0.000306502 |
| _476_ | 10-O-Acetylisocalamendiol | 56.9 | 8.68948 | C_17_H_28_O_3_ | 325.2020894 | 0.000125462 | 0.001426547 |
| _477_ | Phyllostadimer A | 56.6 | 7.55440 | C_42_H_50_O_16_ | 809.3038381 | 0.000000000 | 0.000247314 |
| _478_ | 6,8-Dihydroxy-3-methylisocoumarin | 56.6 | 7.65118 | C_10_H_8_O_4_ | 225.075736 | 0.000007059 | 0.000353829 |
| _479_ | Ceplignan | 56.4 | 6.40063 | C_18_H_18_O_7_ | 327.0874889 | 0.000000000 | 0.000074163 |
| _480_ | (S,E)-Deca-2,9-diene-4,6-diyne-1,8-diol | 56.4 | 4.46215 | C_10_H_10_O_2_ | 207.0658837 | 0.000084147 | 0.000129334 |
| _481_ | 3-Methylcytidine | 56.4 | 0.94547 | C_10_H_15_N_3_O_5_ | 258.1084885 | 0.000000000 | 0.000356303 |
| _482_ | Eupalinolide O | 56.3 | 4.70723 | C_22_H_26_O_8_ | 401.1595147 | 0.000000000 | 0.000131704 |
| _483_ | (R)-a-Terpinyl b-D-glucoside | 56.3 | 12.29578 | C_16_H_28_O_6_ | 361.1873584 | 0.014064504 | 0.000171174 |
| _484_ | trans-2-Undecenoic acid | 56.2 | 8.41478 | C_11_H_20_O_2_ | 229.1442335 | 0.000801330 | 0.000048898 |
| _485_ | Gentianine | 56.1 | 6.99313 | C_10_H_9_NO_2_ | 196.0371797 | 0.000001101 | 0.000057004 |
| _486_ | Acuminoside | 56.1 | 4.97702 | C_21_H_36_O_10_ | 447.2237864 | 0.000000000 | 0.000211956 |
| _487_ | 3,4'-Dihydroxy-3',5'-dimethoxypropiophenone | 56.1 | 5.24620 | C_11_H_14_O_5_ | 225.0765596 | 0.000902517 | 0.004849032 |
| _488_ | N-Acetyltryptamine | 56 | 13.46025 | C_12_H_14_N_2_O | 239.0594312 | 0.003111025 | 0.000085803 |
| _489_ | 8-Amino-7-oxononanoic acid | 55.9 | 12.51757 | C_9_H_17_NO_3_ | 170.1176267 | 0.000277034 | 0.000003829 |
| _490_ | Melilotoside | 55.9 | 4.69660 | C_15_H_18_O_8_ | 325.0929672 | 0.000000000 | 0.000208517 |
| _491_ | Cyclo(L-Leu-trans-4-hydroxy-L-Pro) | 55.7 | 3.98042 | C_11_H_18_N_2_O_3_ | 259.1652278 | 0.000000000 | 0.000128454 |
| _492_ | Sarracenin | 55.7 | 4.69450 | C_11_H_14_O_5_ | 227.0914034 | 0.000254711 | 0.001207813 |
| _493_ | Eurostoside | 55.6 | 6.00025 | C_24_H_28_O_11_ | 491.1564829 | 0.000000000 | 0.000456037 |
| _494_ | 3-O-Ethyl-L-ascorbic acid | 55.6 | 5.29798 | C_8_H_12_O_6_ | 249.0615517 | 0.000069183 | 0.000087616 |
| _495_ | Rutinose | 55.5 | 0.77835 | C_12_H_22_O_10_ | 371.1194631 | 0.000000000 | 0.000301051 |
| _496_ | Salirepin | 55.4 | 5.49627 | C_13_H_18_O_8_ | 267.0862423 | 0.000000000 | 0.000076066 |
| _497_ | Eugenin | 55.4 | 4.64430 | C_11_H_10_O_4_ | 239.0907993 | 0.000279331 | 0.000067438 |
| _498_ | N-Acetyl-alpha-Asp-Glu | 55.3 | 0.97668 | C_11_H_16_N_2_O_8_ | 346.124463 | 0.000000000 | 0.000382088 |
| _499_ | Comanthosid B | 55.3 | 5.87202 | C_23_H_22_O_12_ | 489.1042604 | 0.000000000 | 0.000157946 |
| _500_ | Angelicain | 55.2 | 7.08280 | C_15_H_16_O_6_ | 273.0769254 | 0.000000000 | 0.000035200 |
| _501_ | Obscuraminol F | 55 | 9.21577 | C_16_H_33_NO | 288.2896168 | 0.000804111 | 0.000348779 |
| _502_ | Nandinaside A | 55 | 4.22225 | C_22_H_22_O_10_ | 445.1118963 | 0.000000000 | 0.000119296 |
| _503_ | L-Arginine | 55 | 0.78347 | C_6_H_14_N_4_O_2_ | 175.1190725 | 0.000043822 | 0.000866460 |
| _504_ | Curlignan | 55 | 5.85855 | C_19_H_20_O_7_ | 341.1030799 | 0.000083220 | 0.000276648 |
| _505_ | p-Hydroxymandelic acid | 54.9 | 4.65717 | C_8_H_8_O_4_ | 167.0342991 | 0.000926298 | 0.000098883 |
| _506_ | Cassiachromone | 54.9 | 5.96468 | C_13_H_12_O_4_ | 233.0808343 | 0.000000000 | 0.000074385 |
| _507_ | Alyxialactone | 54.8 | 5.12920 | C_10_H_16_O_4_ | 445.2082333 | 0.000000000 | 0.000765087 |
| _508_ | 3,4-O-Isopropylidene shikimic acid | 54.8 | 5.10528 | C_10_H_14_O_5_ | 195.0657289 | 0.001347684 | 0.007448748 |
| _509_ | Ethyl (E)-3'-hydroxy-4'-methoxycinnamate | 54.8 | 4.74745 | C_12_H_14_O_4_ | 267.0874735 | 0.000057673 | 0.000340793 |
| _510_ | 5,7-Dihydroxyphthalide | 54.8 | 4.60372 | C_8_H_6_O_4_ | 211.0244542 | 0.000000000 | 0.000395353 |
| _511_ | Acetosyringone | 54.8 | 5.46855 | C_10_H_12_O_4_ | 195.0657792 | 0.002064533 | 0.002012689 |
| _512_ | Juncuenin D | 54.7 | 6.25678 | C_18_H_18_O_3_ | 327.123884 | 0.000000000 | 0.000066754 |
| _513_ | Dihydrocoriandrin | 54.7 | 6.48137 | C_13_H_12_O_4_ | 277.0717781 | 0.000000000 | 0.000091776 |
| _514_ | N-(1-Carboxy-2-phenylethyl)glutamine | 54.6 | 5.40242 | C_14_H_18_N_2_O_5_ | 275.1039014 | 0.000011180 | 0.000506985 |
| _515_ | griffonilide | 54.6 | 5.27693 | C_8_H_8_O_4_ | 169.0495993 | 0.000358062 | 0.000411099 |
| _516_ | Gly Phe | 54.6 | 3.04797 | C_11_H_14_N_2_O_3_ | 223.1076784 | 0.000000000 | 0.000607922 |
| _517_ | 3 Hydroxycoumarin | 54.5 | 8.13755 | C_9_H_6_O_3_ | 163.0389678 | 0.033804220 | 0.002413285 |
| _518_ | Acetovanillone primeveroside | 54.4 | 4.06305 | C_20_H_28_O_12_ | 459.1512569 | 0.000000000 | 0.000535318 |
| _519_ | Cryptomeridiol | 54.4 | 13.24902 | C_15_H_28_O_2_ | 239.2014337 | 0.000541616 | 0.000017108 |
| _520_ | Frangulin B | 54.3 | 0.78347 | C_20_H_18_O_9_ | 385.0920636 | 0.002544613 | 0.000000000 |
| _521_ | 2-Hydroxypalmitic acid | 54.3 | 11.36163 | C_16_H_32_O_3_ | 336.250888 | 0.001080508 | 0.000003043 |
| _522_ | N-Acetyl-D-Glucosamine | 54.3 | 0.83318 | C_8_H_15_NO_6_ | 204.0867263 | 0.002068067 | 0.000448582 |
| _523_ | Dikegulac | 54.3 | 4.76057 | C_12_H_18_O_7_ | 273.0982439 | 0.000000000 | 0.000043724 |
| _524_ | Apocynol A | 54.2 | 5.11682 | C_13_H_20_O_3_ | 207.1380505 | 0.000023043 | 0.000206401 |
| _525_ | Sinapyl alcohol | 54.2 | 7.26602 | C_11_H_14_O_4_ | 209.0815271 | 0.000021166 | 0.000095418 |
| _526_ | Lupiwighteone | 54.1 | 6.73378 | C_20_H_18_O_5_ | 371.1487285 | 0.000000000 | 0.000133146 |
| _527_ | 5-Hydroxyferulate | 54 | 5.19482 | C_10_H_10_O_5_ | 209.0451426 | 0.000079463 | 0.000050374 |
| _528_ | Octadecanedioic acid | 54 | 10.14693 | C_18_H_34_O_4_ | 313.23854 | 0.001656768 | 0.000462796 |
| _529_ | Quinic acid | 54 | 0.89207 | C_7_H_12_O_6_ | 173.0447929 | 0.000554113 | 0.000761039 |
| _530_ | Methyl 3-hydroxy-4,5-dimethoxybenzoate | 53.9 | 4.22225 | C_10_H_12_O_5_ | 211.0607523 | 0.000525329 | 0.006527691 |
| _531_ | Rosin | 53.9 | 7.27660 | C_15_H_20_O_6_ | 297.1332606 | 0.000000000 | 0.000095827 |
| _532_ | (11E,15Z)-9,10,13-Trihydroxy-11,15-octadecadienoic acid | 53.8 | 10.48498 | C_18_H_32_O_5_ | 327.2177561 | 0.000054314 | 0.000046922 |
| _533_ | N6-(4-Hydroxybenzyl)adenosine | 53.7 | 5.75738 | C_17_H_19_N_5_O_5_ | 338.1232023 | 0.000000000 | 0.000249917 |
| _534_ | norbergenin | 53.6 | 5.52003 | C_13_H_14_O_9_ | 295.0459175 | 0.000000000 | 0.000077390 |
| _535_ | Spathulatol | 53.6 | 7.56695 | C_30_H_34_O_9_ | 583.2187614 | 0.000000000 | 0.000214126 |
| _536_ | 4-Hydroxymethylphenol 1-O-rhamnoside | 53.5 | 7.05905 | C_13_H_18_O_6_ | 251.0923847 | 0.000558596 | 0.000264606 |
| _537_ | Furano(2'',3'',7,6)-4'-hydroxyflavanone | 53.4 | 5.82943 | C_17_H_12_O_4_ | 313.1069553 | 0.000000000 | 0.000068822 |
| _538_ | Salicyclic acid | 53.4 | 3.07107 | C_7_H_6_O_3_ | 171.0652246 | 0.001045216 | 0.001159833 |
| _539_ | Methylpicraquassioside B | 53.3 | 5.77958 | C_20_H_26_O_11_ | 423.129912 | 0.000000000 | 0.000097280 |
| _540_ | 14,15-Dinorcleroda-3,11E-dien-13-one | 53.2 | 12.65493 | C_18_H_28_O | 305.2122434 | 0.001113784 | 0.000009819 |
| _541_ | Ala Tyr | 53.1 | 1.09453 | C_12_H_16_N_2_O_4_ | 253.1182404 | 0.000000000 | 0.000458432 |
| _542_ | Rhodojaponin III | 53 | 8.92433 | C_20_H_32_O_6_ | 389.1948459 | 0.000000000 | 0.000094698 |
| _543_ | Piperidine | 53 | 1.03458 | C_5_H_11_N | 86.09705659 | 0.000245298 | 0.003857399 |
| _544_ | 8,9-Dihydroxy-10-isobutyryloxythymol | 52.9 | 4.89008 | C_14_H_20_O_5_ | 251.1277779 | 0.000000787 | 0.000706086 |
| _545_ | 9-Hydroxy-O-senecioyl-8,9-dihydrooroselol | 52.8 | 5.10528 | C_19_H_20_O_6_ | 389.1243471 | 0.000000000 | 0.000163491 |
| _546_ | Tulipalin A | 52.7 | 16.71213 | C_5_H_6_O_2_ | 99.04458022 | 0.013617012 | 0.000976199 |
| _547_ | Eupalinilide B | 52.7 | 5.31032 | C_20_H_24_O_6_ | 381.1304181 | 0.000000000 | 0.000167307 |
| _548_ | Acetylvanillin | 52.6 | 4.70945 | C_10_H_10_O_4_ | 239.0558501 | 0.000279443 | 0.000327138 |
| _549_ | alpha-Eudesmol | 52.5 | 12.83417 | C_15_H_28_O | 269.2122821 | 0.000408183 | 0.000009124 |
| _550_ | Hyperxanthone E | 52.4 | 5.84538 | C_18_H_16_O_6_ | 327.0875653 | 0.000000000 | 0.000149722 |
| _551_ | Buergerinin B | 52.4 | 4.84292 | C_9_H_14_O_5_ | 185.0809008 | 0.000048473 | 0.001147896 |
| _552_ | Dihydrocarvone | 52.2 | 16.60978 | C_10_H_16_O | 153.1274502 | 0.038573544 | 0.003776272 |
| _553_ | 8-Methoxybonducellin | 52.1 | 5.90937 | C_18_H_16_O_5_ | 357.0981502 | 0.000000000 | 0.000049325 |
| _554_ | Aloeresin D | 52 | 7.21353 | C_29_H_32_O_11_ | 555.1876111 | 0.000000000 | 0.000117933 |
| _555_ | Indoleacetic acid | 51.9 | 5.36332 | C_10_H_8_NO_2_ | 195.0294432 | 0.000006073 | 0.000007283 |
| _556_ | Dihydrojasmone | 51.9 | 13.89928 | C_11_H_18_O | 184.1697135 | 0.000094548 | 0.000000485 |
| _557_ | 2-Methyl-4-pentenoic Acid | 51.9 | 8.66290 | C_6_H_10_O_2_ | 227.1286057 | 0.000497058 | 0.000030650 |
| _558_ | 2-methyl-1-(7-nitro-3,4-dihydro-1H-isoquinolin-2-yl)propan-1-one | 51.9 | 4.57057 | C_13_H_16_N_2_O_3_ | 249.1233474 | 0.000000000 | 0.000169210 |
| _559_ | 2-(2-acetyl-3-hydroxy-5-methoxyphenyl)acetic acid | 51.9 | 4.77442 | C_11_H_12_O_5_ | 223.0608478 | 0.000119700 | 0.000346347 |
| _560_ | nicotinamide | 51.9 | 1.39080 | C_6_H_6_N_2_O | 123.0555395 | 0.000217295 | 0.001340989 |
| _561_ | LINAMARIN | 51.8 | 5.15572 | C_10_H_17_NO_6_ | 246.0982717 | 0.000000000 | 0.000355356 |
| _562_ | (2R)-2-[(2R,5S)-5-[(2S)-2-hydroxybutyl]oxolan-2-yl]propanoic acid | 51.8 | 8.71692 | C_11_H_20_O_4_ | 217.1434514 | 0.001387726 | 0.000281159 |
| _563_ | Xylobiose | 51.8 | 1.89060 | C_10_H_18_O_9_ | 263.0772615 | 0.000114090 | 0.000103905 |
| _564_ | Pterosin B | 51.8 | 2.21638 | C_14_H_18_O_2_ | 263.1026274 | 0.000000000 | 0.000282186 |
| _565_ | Glyceryl monostearate | 51.7 | 14.76140 | C_21_H_42_O_4_ | 400.3420731 | 0.001098232 | 0.000069720 |
| _566_ | Diosbulbin L | 51.6 | 6.49458 | C_19_H_22_O_7_ | 343.118827 | 0.000000000 | 0.000148879 |
| _567_ | Cyclo(Ala-Tyr) | 51.6 | 1.76208 | C_12_H_14_N_2_O_3_ | 279.0986592 | 0.000000000 | 0.000154696 |
| _568_ | Prenyl glucoside | 51.6 | 4.56432 | C_11_H_20_O_6_ | 293.1242418 | 0.000027556 | 0.000150393 |
| _569_ | Cassiaside | 51.6 | 6.71853 | C_20_H_20_O_9_ | 403.1040656 | 0.000000000 | 0.000049767 |
| _570_ | 1-(5-Methyl-3-furanyl)-1,2,3-propanetriol | 51.6 | 1.67227 | C_8_H_12_O_4_ | 153.0548903 | 0.000153291 | 0.000128479 |
| _571_ | Atalantoflavone | 51.6 | 9.19667 | C_20_H_16_O_5_ | 381.0981409 | 0.000000000 | 0.000074259 |
| _572_ | 2-(1-ethylpyrrolo[2,3-b]pyridin-3-yl)-N-(2-fluoro-5-methylphenyl)-1,3-thiazole-4-carboxamide | 51.5 | 4.63285 | C_20_H_17_FN_4_OS | 381.1180349 | 0.000000000 | 0.000099911 |
| _573_ | Linocinnamarin | 51.4 | 4.68392 | C_16_H_20_O_8_ | 385.1143517 | 0.000000000 | 0.000210470 |
| _574_ | Sibiricose A3 | 51.3 | 0.85005 | C_19_H_26_O_13_ | 483.1126898 | 0.016156988 | 0.000000000 |
| _575_ | Hydroxyanigorufone | 51.1 | 8.12482 | C_19_H_12_O_3_ | 289.0857297 | 0.000000000 | 0.000052778 |
| _576_ | Lecocarpinolide J | 51.1 | 7.03310 | C_21_H_28_O_7_ | 427.1512189 | 0.000000000 | 0.000507432 |
| _577_ | Cucurbitacin C | 51 | 5.98945 | C_32_H_48_O_8_ | 561.3400824 | 0.000000000 | 0.000258120 |
| _578_ | 1-O-Caffeoylglucose | 51 | 4.59510 | C_15_H_18_O_9_ | 343.1023359 | 0.000000000 | 0.000081535 |
| _579_ | Thymic acid | 51 | 6.12287 | C_10_H_14_O | 151.1118166 | 0.000465107 | 0.000034181 |
| _580_ | 9-O-Feruloyllariciresinol | 51 | 7.91677 | C_30_H_32_O_9_ | 581.2035639 | 0.000000000 | 0.000123805 |
| _581_ | Sappanone A Dimethyl Ether | 50.9 | 7.16225 | C_18_H_16_O_5_ | 311.0925557 | 0.000000000 | 0.000134518 |
| _582_ | Sutchuenmedin A | 50.9 | 7.26355 | C_33_H_38_O_14_ | 681.2155689 | 0.000000000 | 0.000706444 |
| _583_ | Isohyenanchin | 50.8 | 5.63590 | C_15_H_20_O_7_ | 357.1193662 | 0.000000000 | 0.000274013 |
| _584_ | Loliolide | 50.8 | 6.42930 | C_11_H_16_O_3_ | 197.1172972 | 0.000231523 | 0.000184413 |
| _585_ | Lucidumoside C | 50.8 | 6.09073 | C_27_H_36_O_14_ | 629.2097851 | 0.000000000 | 0.000388381 |
| _586_ | D-Desthiobiotin | 50.7 | 1.31145 | C_10_H_18_N_2_O_3_ | 259.1298697 | 0.000000000 | 0.000231961 |
| _587_ | Ala Thr Leu | 50.7 | 3.53730 | C_13_H_25_N_3_O_5_ | 304.1866078 | 0.000000000 | 0.000204521 |
| _588_ | 3-Amino-3-(4-hydroxyphenyl)propionic acid | 50.7 | 2.51092 | C_9_H_11_NO_3_ | 180.0659701 | 0.000097027 | 0.000061823 |
| _589_ | Dihydrocitflavanone | 50.6 | 8.19163 | C_20_H_20_O_5_ | 385.1296178 | 0.000000000 | 0.000068509 |
| _590_ | 3-(Hydroxymethyl)-2-penten-5-olide | 50.6 | 1.08523 | C_6_H_8_O_3_ | 170.0811885 | 0.000090751 | 0.000185592 |
| _591_ | Acetyl-Tyrosine | 50.6 | 2.34070 | C_11_H_13_NO_4_ | 222.0767474 | 0.000394576 | 0.000335062 |
| _592_ | Homononactinic acid | 50.6 | 8.71618 | C_11_H_20_O_4_ | 215.1284979 | 0.008411921 | 0.000756260 |
| _593_ | Euparin | 50.5 | 4.91417 | C_13_H_12_O_3_ | 249.1121375 | 0.000000000 | 0.000334427 |
| _594_ | 2'-O-Methyluridine | 50.5 | 1.81298 | C_10_H_14_N_2_O_6_ | 257.0778999 | 0.000027168 | 0.000329426 |
| _595_ | 6,8-Di-O-methylcitreoisocoumarin | 50.4 | 6.75863 | C_16_H_18_O_6_ | 613.2282707 | 0.000000000 | 0.000186952 |
| _596_ | Isoleucyl-Valine | 50.4 | 4.06080 | C_11_H_22_N_2_O_3_ | 231.1703204 | 0.000000000 | 0.002268419 |
| _597_ | Cyclo(Leu-Pro) | 50.3 | 5.73440 | C_11_H_18_N_2_O_2_ | 211.1441304 | 0.000043560 | 0.000119584 |
| _598_ | Asp Ile | 50.2 | 2.82087 | C_10_H_18_N_2_O_5_ | 247.1287959 | 0.000000000 | 0.000162144 |

**Table S2 BTL added substances (fragmentation score ≥ 80 )**

| **Compound N_0_.** | **Identification** | **Formula** | **m/z** | **Retention time (min)** |
| --- | --- | --- | --- | --- |
| **Flavonoids** | | | | |
| 1 | Vicenin II | C_27_H_30_O_15_ | 593.1519 | 4.95020 |
| 2 | Vicenin III | C_26_H_28_O_14_ | 565.1555 | 5.31200 |
| 3 | Calealactone B | C_21_H_26_O_9_ | 403.14 | 6.65315 |
| 4 | Epmedin C | C_39_H_50_O_19_ | 821.289 | 6.11628 |
| 5 | Jacein | C_24_H_26_O_13_ | 567.1361 | 5.04040 |
| 6 | Yukovanol | C_20_H_18_O_6_ | 387.1436 | 6.55158 |
| **Isoflavonoids** | | | | |
| 7 | Phomopsinone D | C_12_H_16_O_5_ | 273.1332 | 4.27118 |
| 8 | Torosachrysone 8-O-beta-gentiobioside | C_28_H_36_O_15_ | 611.1988792 | 5.12920 |
| 9 | Isoschaftoside | C_26_H_28_O_14_ | 563.1412 | 5.32323 |
| 10 | Norwogonin-8-O-glucuronide | C_21_H_18_O_11_ | 445.0781 | 5.40242 |
| **Amino acid** | | | | |
| 11 | D-Tryptophan | C_11_H_12_N_2_O_2_ | 203.0821 | 3.86757 |
| 12 | Glycyltyrosine | C_11_H_14_N_2_O_4_ | 239.1026 | 1.07430 |
| 13 | Ala Glu Leu | C_14_H_25_N_3_O_6_ | 332.1816 | 4.00308 |
| 14 | Gly Leu | C_8_H_16_N_2_O_3_ | 187.1082 | 1.99107 |
| 15 | Asp Ile Phe | C_19_H_27_N_3_O_6_ | 394.1973 | 5.73440 |
| 16 | N-Acetyl-tryptophan | C_13_H_14_N_2_O_3_ | 245.0931 | 5.83210 |
| 17 | Ser Leu Ile | C_15_H_29_N_3_O_5_ | 332.2179 | 5.15377 |
| 18 | Asp Phe | C_13_H_16_N_2_O_5_ | 281.1132 | 4.09692 |
| 19 | Ile Leu Glu | C_17_H_31_N_3_O_6_ | 374.2285 | 4.11945 |
| **Phenolic compounds** | | | | |
| 20 | 2-methoxy-4-[3-[3-(trifluoromethyl) anilino imidazo [1,2-a] pyrimidin-2-yl] phenol | C_20_H_15_F_3_N_4_O_2_ | 401.121 | 6.47733 |
| 21 | 9-O-Feruloyl-5,5'-dimethoxylariciresinol | C_32_H_36_O_11_ | 641.2248 | 7.34353 |
| 22 | (-)-Syringaresinol di-O-glucoside | C_34_H_46_O_18_ | 741.2625 | 5.23390 |
| **Terpenoid** | | | | |
| 23 | Fibleucin | C_20_H_20_O_6_ | 357.1331 | 7.52072 |
| 24 | Morindin | C_26_H_28_O_14_ | 545.1305171 | 5.58412 |
| 25 | Secologanic acid | C_16_H_22_O_10_ | 419.1197001 | 1.20513 |
| 26 | Paeonilactone C | C_17_H_18_O_6_ | 363.1087108 | 5.55717 |
| 27 | Oleuropein aglycone | C_19_H_22_O_8_ | 377.1243539 | 5.92250 |
| 28 | (+)-Abscisic acid | C_15_H_20_O_4_ | 263.1289 | 7.18863 |
| 29 | Ciwujiatone | C_44_H_50_O_18_ | 867.307 | 8.07605 |
| 30 | Zeylenol | C_21_H_20_O_7_ | 417.1543 | 6.46408 |
| **Glycoside** | | | | |
| 31 | Forsythenside A | C_22_H_26_O_10_ | 451.16 | 7.22663 |
| 32 | Sibiricose A5 | C_22_H_30_O_14_ | 517.1565335 | 4.73443 |
| 33 | 3-Deoxyguanosine | C_10_H_13_N_5_O_4_ | 266.0895 | 1.48558 |
| 34 | N2, N2-Dimethylguanosine | C_12_H_17_N_5_O_5_ | 310.1157 | 3.71282 |
| 35 | Eugenol gentiobioside | C_22_H_32_O_12_ | 509.1648 | 4.89858 |
| 36 | Acanthoside B | C_28_H_36_O_13_ | 579.2091 | 5.23390 |
| 37 | Orcinol gentiobioside | C_19_H_28_O_12_ | 493.1569 | 1.43515 |
| 38 | Adenosine | C_10_H_13_N_5_O_4_ | 268.1038863 | 1.64648 |
| 39 | Populin | C_20_H_22_O_8_ | 373.1281861 | 7.17878 |
| 40 | Pinoresinol 4-O-glucoside | C_26_H_32_O_11_ | 519.1878 | 6.41438 |
| 41 | 6-O-Feruloylglucose | C_16_H_20_O_9_ | 401.1090681 | 2.31387 |
| 42 | Pinostilbenoside | C_21_H_24_O_8_ | 807.2878662 | 7.86507 |
| 43 | Desrhamnosylmartynoside | C_25_H_30_O_11_ | 507.1862464 | 6.29700 |
| 44 | 7-O-Methylaloeasinol | C_20_H_26_O_9_ | 375.1436 | 6.41752 |
| 45 | Fraxamoside | C_25_H_30_O_13_ | 583.1675 | 5.22100 |
| **Quinone compounds** | | | | |
| 46 | 4'-Hydroxypiptocarphin A | C_21_H_26_O_10_ | 459.1284 | 5.92250 |
| 47 | 2-Hydroxy-3-methoxybenzoic acid glucose ester | C_14_H_18_O_9_ | 295.0813 | 6.16125 |
| 48 | 2-Methoxy-1,4-naphthoquinone | C_11_H_8_O_3_ | 187.0396 | 5.57060 |
| **Others** | | | | |
| 49 | Emodin-1-O-beta-gentiobioside | C_27_H_30_O_15_ | 575.1410392 | 5.52003 |
| 50 | 6'-(p-Hydroxybenzoyl) mussaenosidic acid | C_23_H_28_O_12_ | 495.1512755 | 5.18110 |
| 51 | Loganate | C_16_H_24_O_10_ | 357.1192412 | 4.81058 |
| 52 | Tetillapyrone | C_11_H_14_O_6_ | 284.1128 | 4.17935 |
| 53 | Granatomycin E | C_22_H_22_O_11_ | 507.1148681 | 5.02835 |
